# Motif conservation, stability, and host gene expression are the main drivers of snoRNA expression across vertebrates

**DOI:** 10.1101/gr.277483.122

**Published:** 2023-04

**Authors:** Étienne Fafard-Couture, Pierre-Étienne Jacques, Michelle S. Scott

**Affiliations:** 1 Département de biochimie et de génomique fonctionnelle, Faculté de médecine et des sciences de la santé, Université de Sherbrooke, Sherbrooke, Québec J1E 4K8, Canada;; 2 Centre de recherche du Centre hospitalier universitaire de Sherbrooke (CRCHUS), Sherbrooke, Québec J1H 5N3, Canada;; 3 Département de biologie, Faculté des sciences, Université de Sherbrooke, Sherbrooke, Québec J1K 2R1, Canada

## Abstract

Small nucleolar RNAs (snoRNAs) are structured noncoding RNAs present in multiple copies within eukaryotic genomes. snoRNAs guide chemical modifications on their target RNA and regulate processes like ribosome assembly and splicing. Most human snoRNAs are embedded within host gene introns, the remainder being independently expressed from intergenic regions. We recently characterized the abundance of snoRNAs and their host gene across several healthy human tissues and found that the level of most snoRNAs does not correlate with that of their host gene, with the observation that snoRNAs embedded within the same host gene often differ drastically in abundance. To better understand the determinants of snoRNA expression, we trained machine learning models to predict whether snoRNAs are expressed or not in human tissues based on more than 30 collected features related to snoRNAs and their genomic context. By interpreting the models’ predictions, we find that snoRNAs rely on conserved motifs, a stable global structure and terminal stem, and a transcribed locus to be expressed. We observe that these features explain well the varying abundance of snoRNAs embedded within the same host gene. By predicting the expression status of snoRNAs across several vertebrates, we notice that only one-third of all annotated snoRNAs are expressed per genome, as in humans. Our results suggest that ancestral snoRNAs disseminated within vertebrate genomes, sometimes leading to the development of new functions and a probable gain in fitness and thereby conserving features favorable to the expression of these few snoRNAs, the large remainder often degenerating into pseudogenes.

Vertebrate genomes are shaped by multiple events such as whole-genome duplication, mutations, recombination, and retrotransposition events, which led in the past to several evolutionary highlights such as the vertebrate land invasion (Dehal and Boore 2005; Kuzmin et al. 2022). Protein-coding genes were investigated quite intensively in that matter, whereas noncoding genes received considerably less attention. Small nucleolar RNAs (snoRNAs), a type of noncoding RNA observed in all eukaryotes, are present in multiple copies within genomes (Dieci et al. 2009; Bouchard-Bourelle et al. 2020), hinting that they are subject to similar evolutionary forces as protein-coding genes. In humans, most snoRNAs are embedded within the introns of either protein-coding or noncoding host genes, the remainder being encoded within intergenic regions (Bouchard-Bourelle et al. 2020). It is assumed that their expression depends on their host gene transcription and splicing or on an independent promoter in the case of intronic and intergenic snoRNAs, respectively (Dieci et al. 2009).

snoRNAs can be divided in two classes based on their structure and function: C/D and H/ACA box snoRNAs, which guide, respectively, the 2′-O-methylation and pseudouridylation of target RNAs to which they bind (Kiss 2001; Filipowicz and Pogačić 2002). The most common snoRNA targets are ribosomal RNAs (rRNAs) and small nuclear RNAs (snRNAs), their snoRNA guided modifications being important for the faithful assembly of ribosome and spliceosome (Dupuis-Sandoval et al. 2015). A substantial proportion of snoRNAs remain with no canonical target (they are referred to as orphan snoRNAs), although growing evidence points to new snoRNA targets and functions such as the regulation of pre-mRNA stability and splicing through snoRNA/pre-mRNA interactions in *cis* or in *trans* (Falaleeva et al. 2017; Bergeron et al. 2020; Bratkovič et al. 2020). Therefore, in this work, a snoRNA is considered functional if it is at least transcribed and interacts with a target in a way that it induces a cellular change (e.g., alteration of the level of RNA modification, abundance, splicing, etc.).

Both snoRNA types harbor specific motifs that promote the recruitment of core proteins and RNA-modifying enzymes to the snoRNA, increasing the stability of the resulting ribonucleoprotein complex (snoRNP) (Matera et al. 2007; Kufel and Grzechnik 2019). Indeed, C/D box snoRNAs possess two conserved motifs, the C and D boxes (with respective consensus sequences RUGAUGA and CUGA, in which R is any purine), as well as the less conserved C′ and D′ motifs (same sequences as C and D boxes), all of which attract the binding of the methyltransferase fibrillarin and of core proteins (i.e., SNU13, NOP58, and NOP56) to the snoRNA (Filipowicz and Pogačić 2002; Matera et al. 2007). As for H/ACA box snoRNAs, they are characterized by the H box (denoted by the ANANNA motif, in which N is any nucleotide), which is present in the hinge region between two hairpins and by the ACA motif that is usually located 3 nucleotides (nt) upstream of the snoRNA 3′ end (Ganot et al. 1997; Matera et al. 2007). The H/ACA box snoRNA structure is bound by protein partners such as NHP2, NOP10, and GAR1, as well as the pseudouridine synthase dyskerin (Massenet et al. 2017).

We recently characterized the abundance patterns of snoRNAs across several healthy human tissues using TGIRT-seq (Fafard-Couture et al. 2021), a high-throughput RNA sequencing approach that accurately quantifies both structured and less structured RNAs in the same sample, for instance, snoRNAs and their host gene, thanks to the use of a high-processivity and high-fidelity thermostable reverse transcriptase (Nottingham et al. 2016; Qin et al. 2016). We found that expressed human snoRNAs are either uniformly expressed across tissues or enriched in one or a few tissues (e.g., brain, reproductive tissues, etc.) (Fafard-Couture et al. 2021). These two abundance classes are mainly regulated by the presence or absence of a dual-initiation promoter within the host gene, which, combined with the nonsense-mediated decay (NMD) pathway, allows the uncoupling of snoRNA and host gene expression (Lykke-Andersen et al. 2016; Nepal et al. 2020; Fafard-Couture et al. 2021). Furthermore, we and other groups have shown that the abundance of most snoRNAs does not correlate well with that of their host gene or even between snoRNA copies, with the observation that snoRNAs embedded within the same host gene often vary extremely in terms of abundance (from not expressed to highly abundant) (Warner et al. 2018; McCann et al. 2020; Bergeron et al. 2021; Fafard-Couture et al. 2021). In addition, the fact that most snoRNAs exist in multiple copies within a genome (Dieci et al. 2009; Bergeron et al. 2021) (and sometimes in the range of thousands of copies [Schmitz et al. 2008]) complicates even more our understanding of how and why a given snoRNA is expressed whereas some or most of its copies are not. Overall, these observations highlight that it is by characterizing the whole snoRNA spectrum (both expressed and not expressed snoRNAs) that we will better understand the main determinants of snoRNA expression, which remain to this day still ill-defined.

Current knowledge on the mechanisms modulating snoRNA abundance dates back to more than 20 years ago, when the expression of a few C/D box snoRNAs was shown to depend on a 40- to 50-nt distance between the snoRNA and the branchpoint within its intron (Hirose and Steitz 2001; Hirose et al. 2006). This strict distance range was shown to be crucial (not too close nor too far), as the assembly of these snoRNPs depends on the binding of the helicase AQR (also known as IBP160) at a 33- to 40-nt distance upstream of the branchpoint (Hirose et al. 2006). As for H/ACA box snoRNAs, a study based on 80 of them identified that expressed H/ACA box snoRNAs do not show any preference in intronic location (Richard et al. 2006). In addition, the formation of a terminal stem was observed to be crucial for the biogenesis of a few C/D box snoRNAs, often compensating when the snoRNA is located farther away than the optimal 40- to 50-nt distance from the branchpoint (Hirose and Steitz 2001). Considering that current up-to-date annotation files comprise more than 1500 human snoRNAs that were discovered over the years, it raises the question of whether these previously mentioned mechanisms apply to the majority of snoRNAs.

To identify the main expression determinants of snoRNAs, we first collected for all human snoRNAs more than 30 features related to the snoRNAs themselves or to their genomic context. We also defined their expression status, that is, the binary state of being either expressed or not expressed in human tissues based on our TGIRT-seq data sets. We then trained several machine learning models to predict, based on the collected features, the expression status of each human snoRNA. By interpreting the decisions made by the predictors, this work aims at identifying which features constitute the main drivers of snoRNA expression in humans, as well as in several other vertebrates to which the models were applied. Furthermore, this work seeks to understand the role of these expression drivers in the evolution of snoRNA repertoires across vertebrate genomes.

## Results

### Expanding the characterization of features known to influence snoRNA expression to the whole human snoRNome challenges current knowledge on snoRNA biogenesis

**Figure 1.**
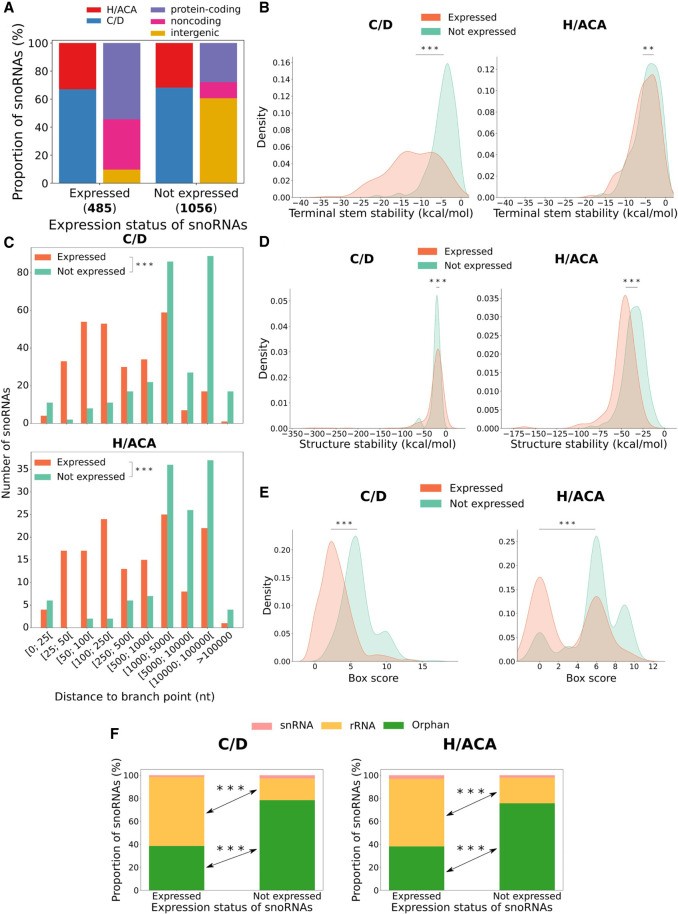
Characterization of human snoRNA features according to their expression status. (*A*) Proportion of expressed and not expressed snoRNAs as a function of the snoRNA type (*left* bar of each pair) and the host gene biotype (*right* bar of each pair). The number of expressed and not expressed snoRNAs are displayed in parentheses *under* the bars. (*B*) Distribution of terminal stem stability (in kcal/mol) for C/D and H/ACA box snoRNAs per expression status. The distributions are statistically different: Mann–Whitney *U* test; (***) *P* < 8 × 10^−79^ and (**) *P* < 0.01, respectively, for C/D and H/ACA box snoRNAs. (*C*) Distribution of the number of expressed and nonexpressed intronic snoRNAs per snoRNA type according to their distance to the branchpoint: Mann–Whitney *U* test; (***) *P* < 6 × 10^−80^ and (***) *P* < 8 × 10^−36^ for C/D and H/ACA box snoRNAs, respectively. (*D*) Distribution of the snoRNA structure stability (in kcal/mol) per expression status and snoRNA type: Mann–Whitney *U* test; (***) *P* < 0.001 and (***) *P* < 2 × 10^−23^ for C/D and H/ACA box snoRNAs, respectively. (*E*) Distribution of snoRNA box score depending on the expression status and snoRNA type: Mann–Whitney *U* test; (***) *P* < 4 × 10^−80^ and (***) *P* < 4 × 10^−19^, respectively, for C/D and H/ACA box snoRNAs. (*F*) Bar charts displaying the proportion of snoRNAs per expression status for C/D (*left* panel) and H/ACA box (*right* panel) snoRNAs according to their target: Fisher's exact test; (***) *P* < 2 × 10^−38^ and (***) *P* < 4 × 10^−15^ for C/D and H/ACA box snoRNAs, respectively.

To define the expression status of all human snoRNAs (i.e., which snoRNAs are expressed or not expressed in humans; see Methods), we used an updated annotation file containing 1541 snoRNAs to reanalyze our published TGIRT-seq samples from seven healthy human tissues (breast, ovary, prostate, testis, liver, brain, and skeletal muscle) (Fafard-Couture et al. 2021). Less than one-third (485/1541, 31.5%) of these snoRNAs are expressed in at least one of these tissues (abundance > 1 transcript per million [TPM]), the majority being C/D box snoRNAs embedded within protein-coding or noncoding host genes (Fig. 1A). Most nonexpressed snoRNAs are also C/D box snoRNAs, but they are mainly located in intergenic regions (Fig. 1A). As previously mentioned, the formation of a terminal stem was shown to be important for the biogenesis of a few C/D box snoRNAs (Xia et al. 1997; Darzacq and Kiss 2000). We hypothesized that a terminal stem could also be formed and be important for H/ACA box snoRNA biogenesis, as their 5′ and 3′ ends are often closely located in snoRNA secondary structural representation (Supplemental Fig. S1; Kalvari et al. 2018). Thus, to verify if this principle applies to all snoRNAs (C/D and H/ACA box snoRNAs), we computed the stability of a potential terminal stem constituted of flanking and internal snoRNA nucleotides for each snoRNA. Expressed C/D box snoRNAs display a significantly more stable terminal stem than their nonexpressed counterparts (Fig. 1B, left panel). Although less stable, we report here that many H/ACA box snoRNA terminal stems could be formed (with stabilities of up to −18.1 kcal/mol) (Fig. 1B, right panel), as seen with C/D box snoRNAs. However, because the terminal stem stability distributions of expressed and nonexpressed H/ACA box snoRNAs are highly similar, it is possible that these potential terminal stems only serve to promote the expression of a handful of H/ACA box snoRNAs. As these terminal stems contain multiple gaps (which hinder direct length count), we also created a terminal stem length score to approximate the length of these terminal stems (see Methods). Consistently, expressed C/D box snoRNAs display significantly higher terminal stem length scores than their nonexpressed counterparts, which is not the case for H/ACA box snoRNAs (Supplemental Fig. S2A). As mentioned earlier, snoRNA distance to the branchpoint was shown to be crucial for the biogenesis of a small number of extensively characterized intronic C/D box snoRNAs but not of intronic H/ACA box snoRNAs (Hirose and Steitz 2001; Richard et al. 2006; Vincenti et al. 2007). To validate these findings across our updated human snoRNA catalog, we calculated the distance to the branchpoint for all snoRNAs. Most expressed C/D and H/ACA box snoRNAs are at least 100 nt away from their intron branchpoint (and sometimes up to >100,000 nt) (Fig. 1C), which is in opposition with what is currently assumed in the literature as an optimal location for C/D box snoRNAs. We also find a significant tendency for expressed snoRNAs to be closer to the branchpoint than their nonexpressed counterparts for both snoRNA types (Fig. 1C). Further investigating, we observe that expressed C/D box snoRNAs embedded close to the branchpoint (≤100 nt) are significantly more likely to target rRNA than expressed C/D box snoRNAs located far from the branchpoint (>100 nt) (Supplemental Fig. S3A). In addition, introns harboring expressed C/D box snoRNAs that are close to the branchpoint are bound markedly more often by AQR (Supplemental Fig. S3B). Expressed C/D box snoRNAs located far from the branchpoint display a more stable structure and less degenerate boxes than those located closer to the branchpoint (Supplemental Fig. S3C,D). This suggests that C/D box snoRNAs located close to the branchpoint are “typical” snoRNAs (i.e., those with a canonical rRNA target and for which their expression depends on AQR), whereas those located farther away are more atypical in their target and display enhanced characteristics to compensate for a suboptimal branchpoint distance. Altogether, these results underline that a stable terminal stem is observed for most expressed C/D box snoRNAs (and also, but to a lesser degree, H/ACA box snoRNAs) and that both types of expressed intronic snoRNAs are mainly located at a greater distance from their intron branchpoint than what is currently assumed as an optimal distance.

As splicing is involved in intronic snoRNA biogenesis (Yang 2015), we also computed the distance between intronic snoRNAs and their upstream and downstream exons, resulting in similar distributions to what is seen with snoRNA distance to the branchpoint (Supplemental Fig. S2B,C). Notably, several host genes contain many introns (up to 147), hinting to a potential wide range of intron lengths in which snoRNAs can be embedded (Supplemental Fig. S4A). Indeed, we find that both types of expressed snoRNAs tend to be embedded in smaller introns than nonexpressed snoRNAs (Supplemental Fig. S2D), suggesting that intronic snoRNA production might be promoted because of smaller introns that facilitate the splicing process. We also observe that expressed intronic snoRNAs are located in introns significantly more downstream in host genes compared with their nonexpressed counterparts (Supplemental Fig. S4B,C). This is in line with the small intron hypothesis, as downstream introns are usually shorter than the first introns in several eukaryotes (Bradnam and Korf 2008; Zhang and Edwards 2012). A significant difference is also found between expressed and nonexpressed intronic snoRNAs according to their intron rank computed from the 3′ end of the gene, highlighting that expressed snoRNAs are preferentially encoded farther away from the 3′ end than nonexpressed snoRNAs (Supplemental Fig. S4D). Taken together, these results indicate that expressed snoRNAs are preferentially encoded within smaller introns, which are located, on average, midway between the first and last introns of host genes, whereas nonexpressed snoRNAs are mainly located in introns close to the 5′ or 3′ ends of host genes.

### snoRNA expression status also varies according to novel features

**Figure 2.**
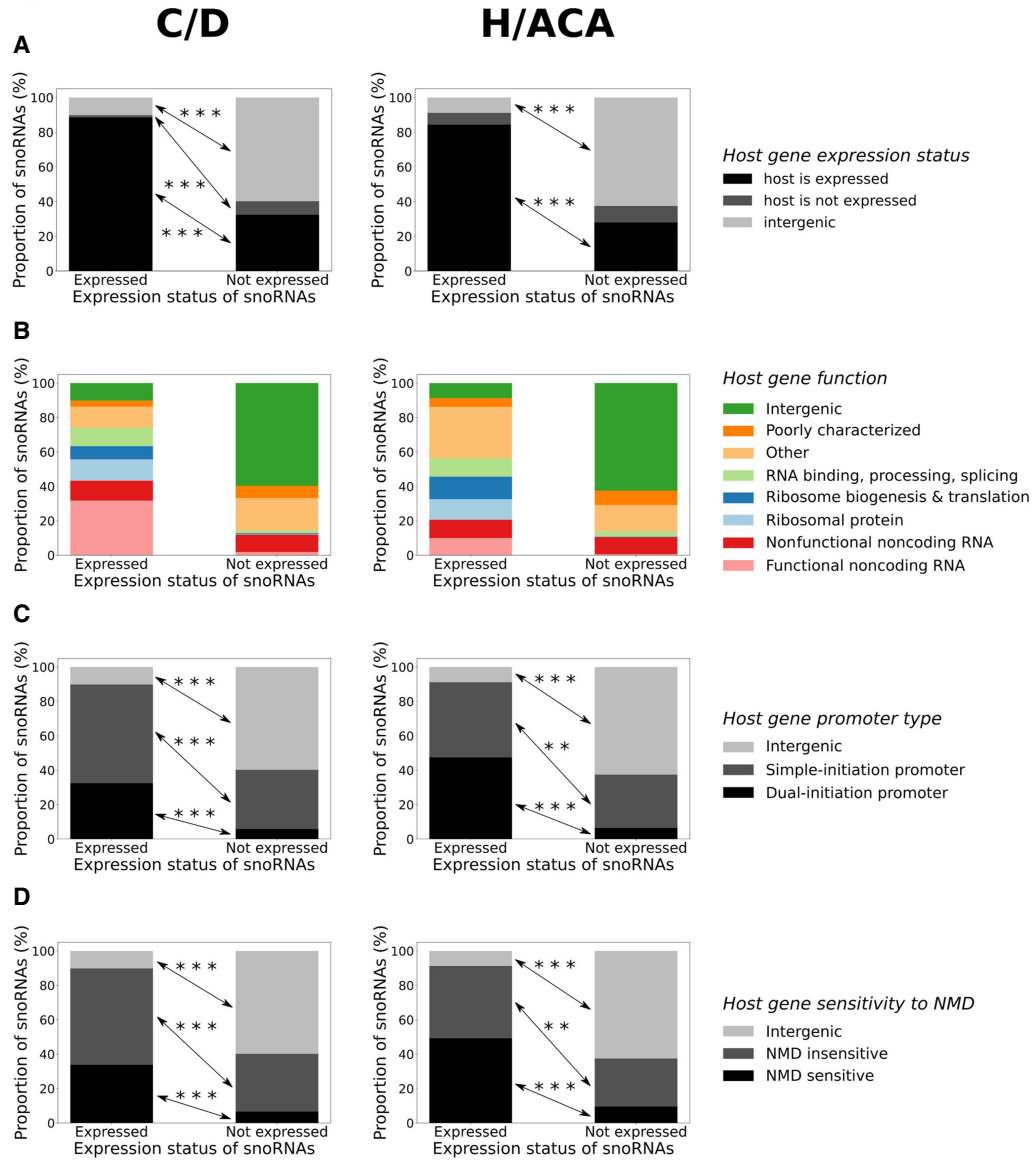
Host gene features vary considerably between snoRNA expression statuses. (*A*–*D*) Proportion of snoRNAs per expression status for C/D (*left* panel) and H/ACA box snoRNAs (*right* panel) according to their genomic context (*A*), their host gene function (*B*), promoter type (*C*), and sensitivity to nonsense-mediated decay (NMD) (*D*). (*A*) Fisher's exact test; (***) *P* < 5 × 10^−6^ and (***) *P* < 2 × 10^−32^, respectively, for C/D and H/ACA box snoRNAs. (*C*) Fisher's exact test; (***) *P* < 4 × 10^−28^ and (***) *P* < 3 × 10^−25^, respectively, for C/D and H/ACA box snoRNAs, and (**) *P* < 0.01. (*D*) Fisher's exact test; (***) *P* < 2 × 10^−27^ and (***) *P* < 6 × 10^−22^, respectively, for C/D and H/ACA box snoRNAs, and (**) *P* < 0.01.

Because snoRNAs are highly structured noncoding RNAs, we hypothesized that their secondary structure stability might influence their expression status. Indeed, expressed H/ACA box snoRNAs display a significantly more stable structure than their nonexpressed counterparts, whereas it is the opposite for C/D box snoRNAs but to a lesser degree (Fig. 1D). To determine whether the absence of conserved motifs within snoRNAs might affect their expression status, we calculated a box score based on the distance to the motif consensus for each snoRNA (see Methods), indicating with a low score that the snoRNA motifs are highly conserved (close to their consensus sequence) and vice versa. We find that expressed snoRNAs of both types display motifs that are significantly more conserved than their nonexpressed counterparts (Fig. 1E), suggesting that these expressed snoRNAs might be functional as they could bind their core proteins and enzymes. Indeed, most expressed C/D and H/ACA box snoRNAs possess a canonical rRNA target, which is significantly less the case for the nonexpressed snoRNAs (Fig. 1F). Nonetheless, 211 nonexpressed snoRNAs display a canonical rRNA target, out of which >87% have at least another snoRNA copy, suggesting that these snoRNAs are nonexpressed copies of snoRNAs with identifiable rRNA targets. Finally, we wondered whether the host gene expression level could be an indicator of snoRNA expression status. Because it was shown by several groups that the exact abundance level of most snoRNAs does not correlate with that of their host gene (Boivin et al. 2018; Warner et al. 2018; McCann et al. 2020; Fafard-Couture et al. 2021), we decided to use a binary approach to define the host gene expression level (i.e., either expressed or nonexpressed; see Methods). Most expressed snoRNAs are produced from an expressed host gene (i.e., detected in our TGIRT-seq data sets), which is significantly less the case for nonexpressed snoRNAs that are mainly encoded within intergenic regions or within host genes that are not expressed (i.e., not detected in our TGIRT-seq data sets) (Fig. 2A). Further investigating host gene characteristics, we discovered that those encoding expressed snoRNAs are enriched with functions such as ribosomal protein, ribosome biogenesis and translation, RNA binding/processing/splicing, and functional noncoding RNA (Fig. 2B). In addition, host genes of expressed snoRNAs are significantly more prone to harbor a dual-initiation promoter and be subject to NMD than are host genes of nonexpressed snoRNAs (Fig. 2C,D). Overall, these results constitute a comprehensive catalog of human snoRNA features and suggest that a diverse combination of features explain the expression status of snoRNAs.

### All models predicting snoRNA expression status are highly performant and concordant

**Figure 3.**
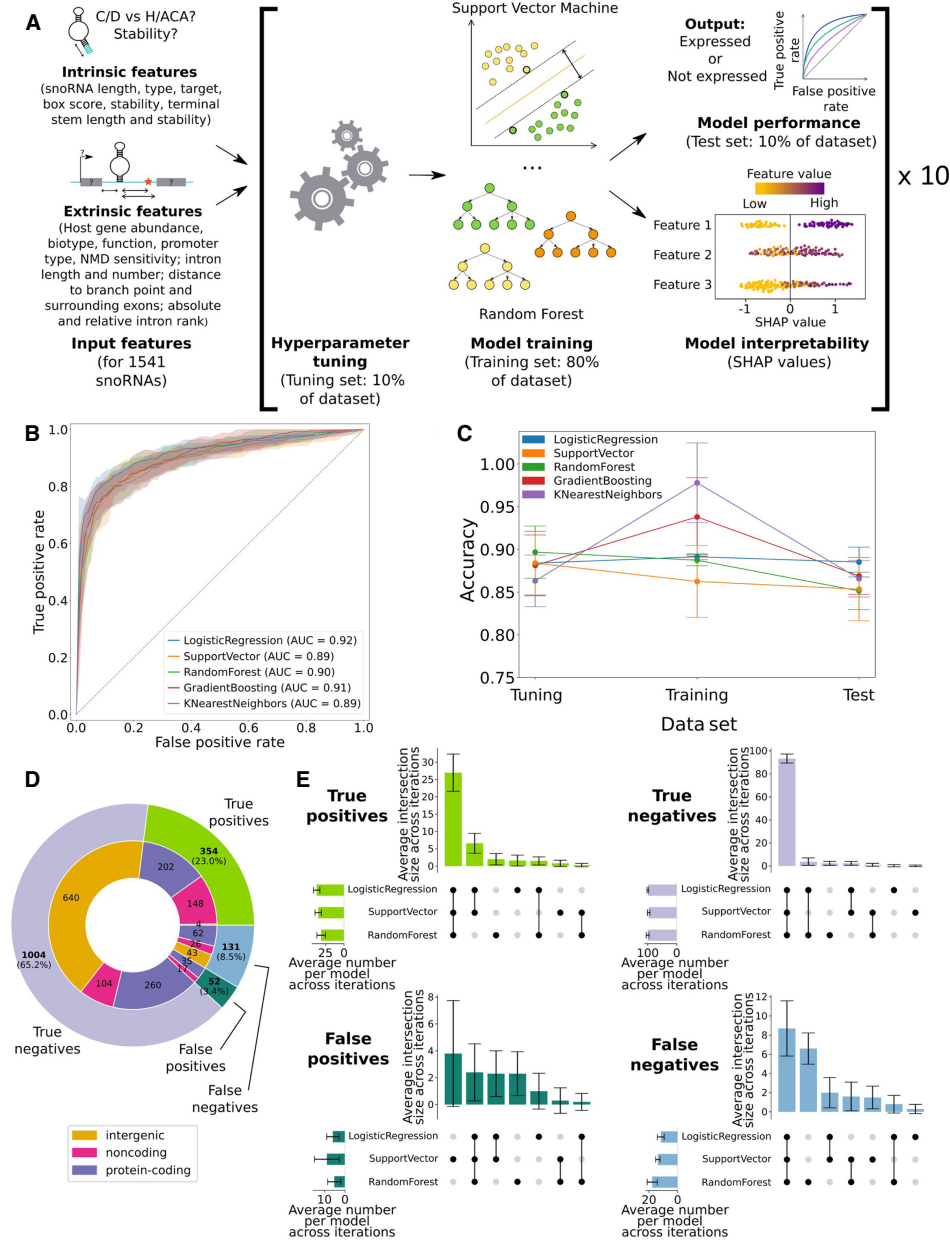
All models predicting snoRNA expression status are highly performant and concordant. (*A*) Features were collected for all human snoRNAs and used to optimize the hyperparameters of each model. The optimized models were then trained (only the support vector machine and random forest classifiers are represented) and tested, and their decisions were interpreted using Shapley additive explanations (SHAP values). This process was repeated across 10 randomized data set splits (iterations), ensuring one expression status prediction for each snoRNA. (*B*) Receiver operating characteristic (ROC) curves showing the average true- and false-positive rates of each model on the test sets (the colored areas around each curve represent ±1 SD across the 10 iterations). The average area under the curve (AUC) is shown for each classifier. (*C*) Average accuracy (±SD) of each model on the tuning, training, and test sets across the 10 iterations. (*D*) Distribution of the number of snoRNAs based on their predicted value. A snoRNA is considered as, for example, a true positive when at least two of the three selected models predict it as such. (*E*) Average intersection of predictions (±SD) between all models for the different prediction types across the 10 iterations.

Based on the several intrinsic and extrinsic snoRNA features described above, we sought to identify the main determinants of human snoRNA expression using a machine learning approach (Fig. 3A). We chose this avenue over classical statistical approaches for several reasons, including the fact that machine learning is generally better at capturing complex relationships between variables, especially when dealing with a high number of input features relative to the number of examples (Bzdok et al. 2018). Consequently, we optimized, trained, and tested five classifiers based on different algorithms (logistic regression, support vector machine, random forest, *k*-nearest neighbors, and gradient boosting) to predict the expression status of snoRNAs following a stratified nested 10-fold cross-validation approach. Doing so, a total of 50 models were thereby trained to predict the expression status of the snoRNAs present in their respective test set, ensuring that each of the 1541 human snoRNAs had its expression status predicted once per model type across the 10 different test sets (Fig. 3A; Supplemental Fig. S5). All the models show high performance and stability across the different iterations, as shown by the high area under the curve (AUC) of their respective receiver operating characteristic (ROC) curve (AUC between 0.89 and 0.92) and the narrow-colored areas, which represent the variability in the predictions across iterations (Fig. 3B). To further evaluate the performance of the different classifiers, the average prediction accuracy across iterations was computed for all models on the tuning, training, and test sets (Fig. 3C). Of note, the gradient boosting and *k*-nearest neighbors show enhanced and diminished accuracy on the training and test sets, respectively, which is a hallmark of overfitting. We thus discarded these classifiers and selected the logistic regression, support vector machine, and random forest models for the rest of our analyses, because they showed high prediction accuracy and stability across the different data sets (Fig. 3C). To obtain the final predicted expression status per snoRNA, we chose an ensemble approach in which the final prediction corresponds to the predicted expression status with the most occurrences across the three selected models. Expectedly, the number of true positives (TPs) and true negatives (TNs) greatly surpassed the number of false positives (FPs) and false negatives (FNs; with an overall specificity and a sensitivity of, respectively, 95% and 73%), where most of the TPs are intronic snoRNAs whereas the vast majority of TNs are encoded in intergenic regions (Fig. 3D). Moreover, the selected models not only are highly accurate but also show high concordance between their predictions, as the three models predict in majority the same snoRNAs as TPs (76% of all TPs), TNs (93% of all TNs) and FNs (66% of all FNs) across iterations (Fig. 3E). Altogether, these results indicate that the selected logistic regression, support vector machine, and random forest models are highly accurate and concordant at predicting snoRNA expression status.

### snoRNA expression status is governed by the conservation of their box sequences, their stability, and their host gene expression status

**Figure 4.**
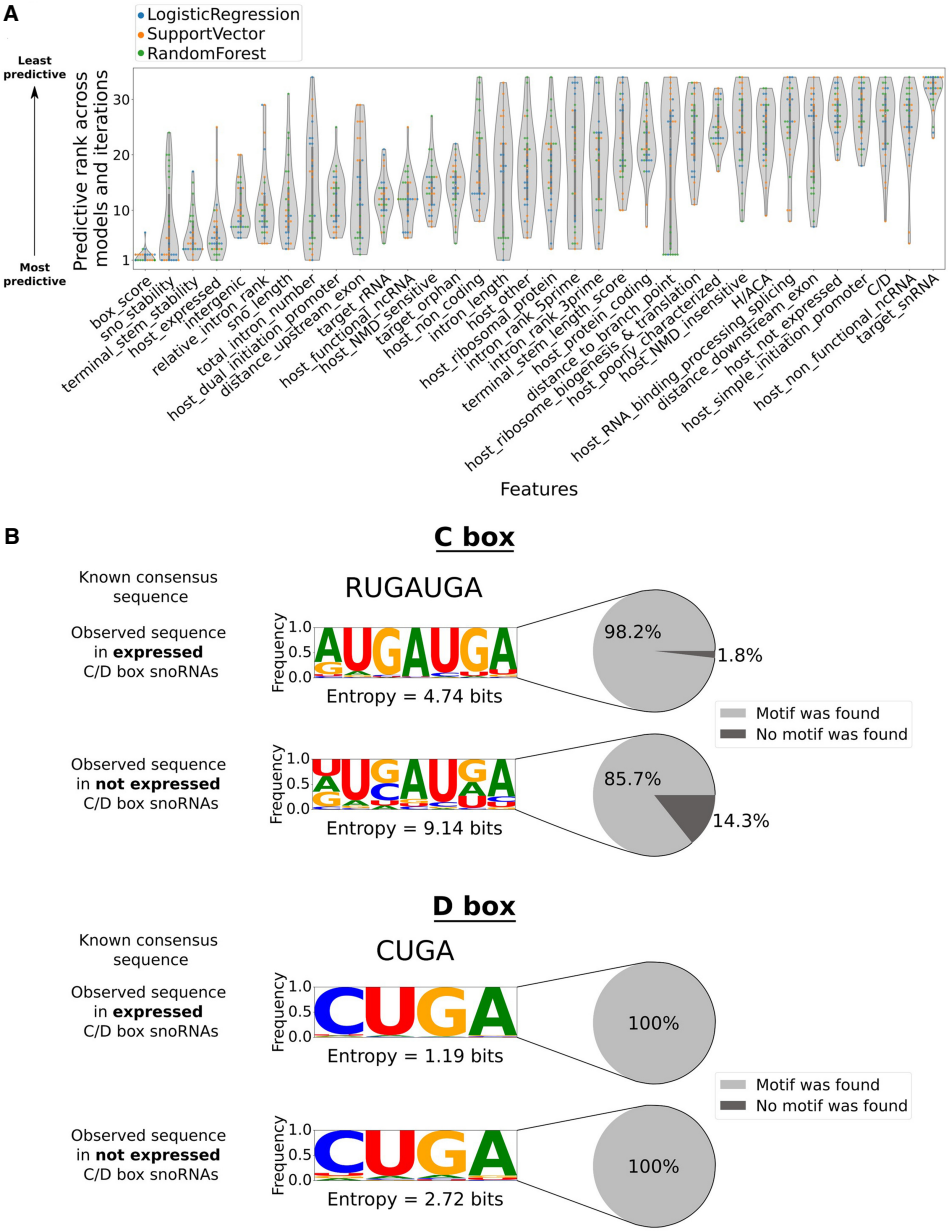
Box sequence conservation, snoRNA stability, and host gene expression level govern the snoRNA expression status. (*A*) Distribution of the predictive rank of each input feature across all selected models and iterations. (*B*) Frequency logos of the observed C (*top* panel) and D (*bottom* panel) motifs in expressed and nonexpressed C/D box snoRNAs (where R is a purine). The cumulative Shannon entropy (sum of the entropy per nucleotide) is shown for each logo, as well as the proportion of snoRNAs in which a motif could be found.

As our classifiers showed high performance, interpreting their predictions was the next logical step in order to gain insight into the main features regulating snoRNA expression status. Based on Shapley additive explanations (SHAP values) (Lundberg and Lee 2017) applied to all human snoRNAs, a predictive rank was computed for all features across models and iterations (see Methods). Of note, the box score is consistently present in the topmost predictive features of all models, highlighting its generalized importance for the models’ decisions (Fig. 4A). SnoRNA structure, terminal stem stability, and the expression status of the host gene are the three other most predictive features, although having more rank variations between model types (Fig. 4A). Most of the remaining features show predictive rank distributions with a small range of values and an increased median, indicating that most models agree at defining these features as less important for their prediction (Fig. 4A). In addition, a few feature distributions such as the distance to the branchpoint, intron length, and total intron number display a wide breadth of predictive ranks, ranging sometimes from most to least predictive across models and iterations (Fig. 4A). Thus, there is not a clear consensus between models with regard to the importance of these features, underlining their centered position on the predictive spectrum between the clearly important and clearly unimportant features. Further analyzing feature importance (using again SHAP values) but this time separately for C/D and H/ACA box snoRNAs, we find no remarkable difference between both snoRNA types according to their feature predictive ranks (Supplemental Figs. S6, S7), suggesting that they share the same expression determinants. Because only a handful of reliable expression data sets comprising both the snoRNA and their host gene abundance is available in the literature, we tested the performance of our predictors using independent and publicly available data to infer host gene expression status. Using either a subset of the Genotype-Tissue Expression (GTEx) project (Lonsdale et al. 2013) matching the tissue composition of our TGIRT-seq data set or the same number of unmatched tissues (both highly concordant regarding the host gene expression status), we find that our models display a highly comparable performance to what is achieved using TGIRT-seq data sets to define host gene level (Supplemental Figs. S8, S9). We thus conclude that the chosen source of host gene abundance is not crucial in our analyses and therefore can be substituted without affecting markedly the predictive performance.

Considering the widespread importance of the box score in predictions across models, we investigated in further detail how this feature might correlate with snoRNA expression status. We find that a higher proportion of expressed C/D box snoRNAs harbors an identifiable C box that is more similar to the known consensus sequence than nonexpressed C/D box snoRNAs, which show significantly more degenerate C motifs when one could be found (with especially great variation at the two G positions in the motif; Kolmogorov–Smirnov test, [*] *P* < 0.05) (Fig. 4B, top panel). We also observe significant, yet much less pronounced, motif degeneration for D boxes (Kolmogorov–Smirnov test, [*] *P* < 0.05) (Fig. 4B, bottom panel). Similarly, slightly more conserved C′ and D′ motifs are found within expressed C/D box snoRNAs compared with their nonexpressed counterparts (Supplemental Fig. S10). In parallel, only 19.3% of nonexpressed H/ACA box snoRNAs display a H motif compared with 55.6% of expressed H/ACA box snoRNAs (Supplemental Fig. S11, top panel). Likewise, the ACA motif is found less often in nonexpressed H/ACA box snoRNAs than in their expressed counterparts (respectively, 68.5% and 89.4% of these snoRNAs) (Supplemental Fig. S11, bottom panel). By reanalyzing available cross-linking and immunoprecipitation data sets (eCLIP and PAR-CLIP) of different snoRNP proteins, including dyskerin, fibrillarin, NOP56, and NOP58 (Kishore et al. 2013; Van Nostrand et al. 2020), we find that expressed snoRNAs are significantly more bound by their respective protein partners than are nonexpressed snoRNAs (Supplemental Fig. S12), underlining the crucial role of motif conservation in snoRNP formation. Overall, these results suggest that box sequence conservation (especially the C, H, and ACA motifs), snoRNA stability (global structure and at the terminal stem level), and host gene expression level are the principal expression determinants of human snoRNAs, regardless of the snoRNA type.

### The predictors elucidate the differing expression status of snoRNAs embedded in the same host gene and identify potential functionally relevant snoRNAs among poorly characterized ones

**Figure 5.**
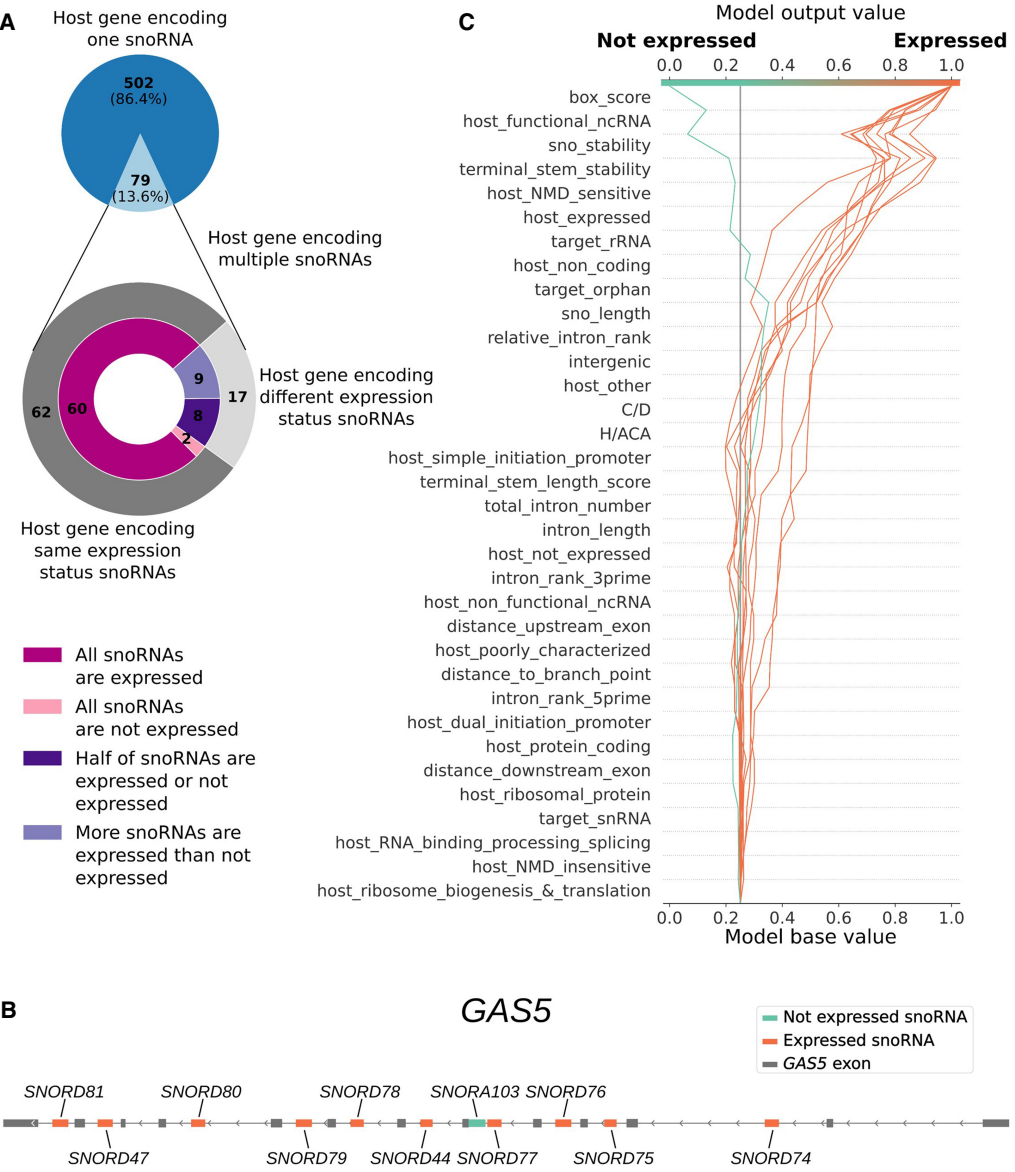
snoRNAs encoded within the same host gene, such as *GAS5*, can differ in expression status owing to their intrinsic features. (*A*) Distribution of the number of host genes encoding one or multiple snoRNAs within the same gene (*top* panel) and their corresponding expression status (*bottom* panel, outer layer) and consistency of expression status (*bottom* panel, inner layer). (*B*) Representation of the host gene *GAS5* (exons shown as gray boxes) that encodes 10 expressed snoRNAs (orange boxes) and one nonexpressed snoRNA (turquoise box). (*C*) Decision plot of the support vector machine classifier showing the relative contribution (SHAP value) of each feature in the decision process of predicting the expression status of each *GAS5* embedded snoRNAs (where each individual line traced toward either the “expressed” or “not expressed” output represents one snoRNA).

In vertebrates, several host genes harbor multiple snoRNAs in different introns, and often these snoRNAs vary in terms of expression status. In humans, 79 (13.6%) of the 581 host genes encode multiple snoRNAs (Fig. 5A). The vast majority of these snoRNAs are present within their host gene with not more than one or two other snoRNAs, most of which are accurately predicted as expressed or not expressed by our classifiers (Supplemental Fig. S13). Out of the 79 host genes encoding multiple snoRNAs, 17 are in the situation in which their embedded snoRNAs vary in expression status, with expressed snoRNAs being always either equal in number or in majority compared with the nonexpressed snoRNAs embedded within their host gene (Fig. 5A).

As SHAP values provide not only a global overview of feature importance (as shown in Fig. 4A) but also a local perspective, it allows us to scrutinize at the snoRNA level which features are important for a given prediction. A compelling example we identified is the host gene *GAS5*, which encodes a tumor-suppressor long noncoding RNA. This host gene harbors 11 intronic snoRNAs, 10 being expressed and one not expressed in human tissues (Fig. 5B; Fafard-Couture et al. 2021). Notably, both the support vector machine and logistic regression classifiers accurately predicted the expression status of all these snoRNAs, allowing us to interpret all their individual predictions. As shown in the decision plot of the support vector machine classifier, *SNORA103*’s prediction to be *not expressed* is mainly influenced by the snoRNA box score, structure stability, target (orphan and not rRNA), and terminal stem stability (Fig. 5C, turquoise curve). This suggests that the unstable structure and terminal stem of this snoRNA and its degenerate motifs hinder its expression (Supplemental Table S1). Conversely, the 10 other *GAS5*-embedded snoRNAs’ predictions to be *expressed* are influenced positively by the box score, terminal stem stability, snoRNA target, and several host gene-related features (i.e., the fact that *GAS5* is expressed, functional, noncoding, and subject to NMD) (Fig. 5C, orange curves; Supplemental Table S1). This suggests that the expression of these 10 snoRNAs is greatly favored because *GAS5* is a functionally important lncRNA that is thereby expressed in humans and because each of these 10 snoRNAs possesses conserved motifs and a stable terminal stem (Supplemental Table S1). Of note, all of the mentioned host gene features similarly disfavor *SNORA103*’s *not expressed* prediction, but not enough to surpass the strongest and most important features in that prediction (snoRNA stability and box score). Expectedly, we reach similar conclusions based on the interpretation of the logistic regression classifier predictions on the same *GAS5*-embedded snoRNAs (Supplemental Fig. S14).

Another interesting case that we investigated is that of FP snoRNAs, that is, snoRNAs predicted to be expressed but that are actually not expressed in our TGIRT-seq data sets. More than 95% of the FPs are embedded within an expressed host gene, resembling closely the host gene expression level of TPs, but not TNs (Supplemental Fig. S15A). These FP snoRNAs also show, in general, significantly higher abundance than the TNs (but still lower than the expression status threshold we used) (Supplemental Fig. S15B), hinting that these snoRNAs have at least the potential to be expressed. One of these FPs is the C/D box snoRNA *SNORD86*, which was previously shown to regulate the splicing and expression level of its host gene (NOP56, a C/D box snoRNA core protein) through conformational changes and core protein trapping in HEK293 cells (Lykke-Andersen et al. 2018). *SNORD86*’s prediction to be expressed is greatly influenced by the presence of conserved motifs, the formation of a stable structure and terminal stem, and because it is encoded within an expressed, NMD-sensitive host gene that is functionally important in ribosome biogenesis (Supplemental Fig. S15C). Considering that our TGIRT-seq data sets only included seven healthy tissues, we reprocessed samples coming from the universal human reference RNA (HumanRef; a pool from 10 cell lines) that were also sequenced using a TGIRT-seq approach (Nottingham et al. 2016). We find that *SNORD86* has an average abundance level of 2.88 TPM in these samples (Supplemental Fig. S15D). In fact, nine of the 52 FP snoRNAs (17%) are considered as expressed (>1 TPM) in these samples (Supplemental Fig. S15D), indicating that these snoRNAs, which were singled out by our classifiers, might be expressed and functional (i.e., interact with a target RNA and/or protein[s] to induce a cellular change) in other tissues that have yet to be analyzed by TGIRT-seq. Overall, these results indicate that our models can explain the differing expression status of snoRNAs encoded within the same host gene and can identify potential functionally relevant snoRNAs even if they were not detected in our initial data sets.

### The models can also accurately predict snoRNA expression status of other species such as the mouse

**Figure 6.**
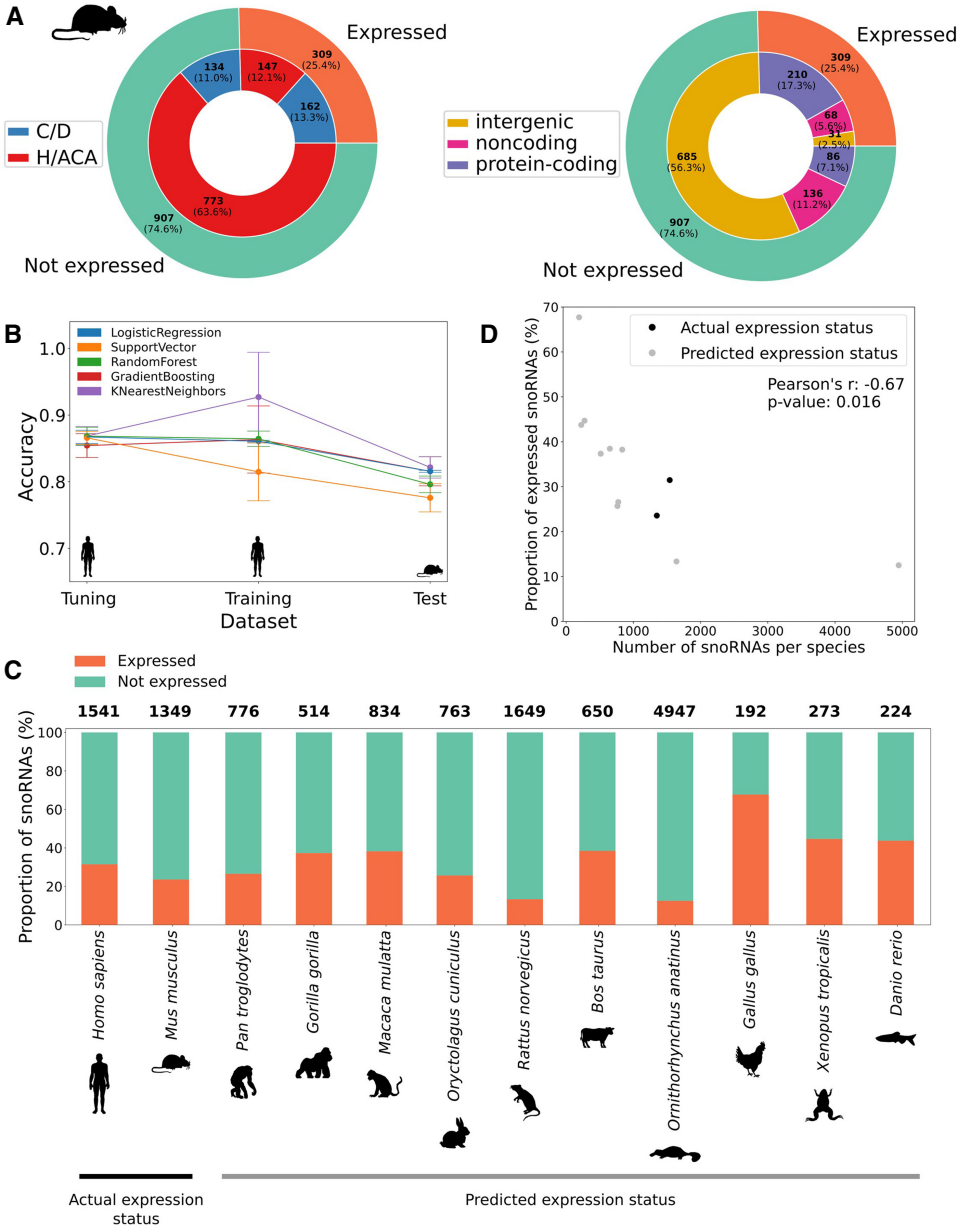
snoRNA expression status prediction in mouse and other vertebrates identifies a conserved low proportion of expressed snoRNAs. (*A*) Distribution of the number of snoRNAs per expression status (*outer* circle) according either to their type (*inner* circle in *left* panel) or genomic location (*inner* circle in *right* panel). (*B*) Average accuracy (±SD) of all models using only the top four features (box_consensus_ score, sno_stability, terminal_stem_stability, and host_expressed) on the tuning (10% of human snoRNAs), training (90% of human snoRNAs), and test (all mouse snoRNAs) sets across five random iterations of tuning/training data sets. (*C*) Proportion of expressed snoRNAs in humans and the mouse (actual expression status based on TGIRT-seq data sets) and for several vertebrate species (predicted expression status). The total number of snoRNAs per species is shown on *top* of each bar. (*D*) Proportion of expressed snoRNAs as a function of the total number of snoRNAs encoded within a species genome. The proportion of expressed snoRNAs is actually measured by TGIRT-seq for human and mouse; all other proportions are predicted using the logistic regression expression status predictor.

To determine if the identified snoRNA expression determinants could be extended to other species, we first reprocessed TGIRT-seq data sets that were recently generated in mouse (*Mus musculus*) embryonic stem cells (mESCs) (McCann et al. 2020). Because these data sets contained only small RNAs, we used an independent RNA-seq data set from 19 different mouse tissues (Shen et al. 2012) to define host gene expression status (previously shown as an appropriate alternative) (Supplemental Figs. S8, S9). Conversely to the human snoRNome, annotated mouse snoRNAs are mostly of the H/ACA box type (Fig. 6A). However, the proportion of expressed snoRNAs stays in similar range between mouse (25.4%) and human (31.5%), with a majority of intronic C/D box snoRNAs constituting the expressed snoRNA pool and a majority of intergenic snoRNAs constituting the nonexpressed pool in both species (Figs. 6A, 1A). Mouse H/ACA box snoRNA feature distributions are quite similar to the human ones, whereas C/D box snoRNAs show reversed tendencies for the box score, terminal stem stability, and host gene expression status (Figs. 1B,D,E, 2A; Supplemental Fig. S16). These reversed trends are nonetheless explainable by 204 nonexpressed C/D box snoRNAs encoded within the same *Snhg14* host gene. Indeed, out of these 204 snoRNAs, 81 display the exact same terminal stem stability (−15.7 kcal/mol) and almost the same box score (most being equal to one), thus driving the feature distributions around these values (Supplemental Fig. S16). It should be noted, however, that a limited diversity of tissues was available for consideration in the mouse, and many such snoRNAs currently defined as nonexpressed in the mouse might actually be expressed in tissues not yet considered by TGIRT-seq.

Considering that the most predictive features used by the models are easy-to-collect snoRNA features and that most of the host gene features are generally less well characterized in the mouse than in humans, we considered simplifying our predictors in order to apply them more conveniently to other species. Human snoRNA expression status was therefore predicted using as input either only the box score, the top three most predictive features (box score, global structure, and terminal stem stability), or the top four (with the addition of the host gene expression status). The models trained with the top four features display an equivalent high performance that is comparable to what is achieved with the complete set of features, which was not the case for the two other simplified models (Supplemental Fig. S17; Fig. 3B,C). Using these top four features, the same five types of models were tuned and trained using, respectively, 10% and 90% of all human snoRNAs across five iterations and tested on mouse snoRNAs. The resulting performance on mouse snoRNAs is similar to the one obtained using all features on human snoRNAs (Figs. 6B, 3C). As the logistic regression classifier showed the highest accuracy and stability of prediction across iterations without overfitting, its best iteration was chosen for further predictions. As expected, it predicts a high number of TPs and TNs composed mostly of intronic and intergenic snoRNAs, respectively, with a specificity of 85% and a sensitivity of 69% (Supplemental Fig. S18).

Finally, we used this model to predict the expression status of snoRNAs across a wide breadth of vertebrate species, ranging from the chimpanzee (*Pan troglodytes*) to the zebrafish (*Danio rerio*) using publicly available transcriptomic data from the Bgee database (Bastian et al. 2021). Of note, except for the chicken (*Gallus gallus*), all of these vertebrate species show a smaller proportion of predicted expressed snoRNAs compared with the predicted nonexpressed snoRNAs (ranging from ∼13% to ∼45% of predicted expressed snoRNAs), as we observe in humans and the mouse based on TGIRT-seq data sets (Fig. 6C). Moreover, we find a significant anticorrelation between the number of annotated snoRNAs in a genome and the proportion of expressed snoRNAs in that species (Pearson's r = −0.67 and [*] *P* < 0.05) (Fig. 6D). Taken together, these results suggest that our machine-learning based approach can be applied accurately to species other than human and that only a small subset of currently annotated snoRNAs needs to be expressed across vertebrates.

## Discussion

In the present work, by assembling a comprehensive catalog of more than 30 snoRNA features from which predictive models could learn, we greatly expand the understanding of the main determinants of snoRNA expression in vertebrates. By reprocessing TGIRT-seq data sets using up-to-date annotations, we find that only 31.5% of snoRNAs are expressed in healthy human tissues, most of them being intronic C/D box snoRNAs (Fig. 1A). Based on the interpretation of the models’ predictions, our study corroborates previous reports supporting the importance of the terminal stem (Figs. 1B, 4A; Supplemental Fig. S2A), identifying, in addition, the stability of the global secondary structure of the snoRNA (Figs. 1D, 4A), the conservation of the sequence motifs (Figs. 1E, 4A,B; Supplemental Figs. S10, S11), and the expression status of the host gene (Figs. 2A, 4A) as the four most important expression determinants (Fig. 4A; Supplemental Fig. S17). Of note, the distance between a snoRNA and the branchpoint in its encoding intron, which is currently assumed in the literature to be a crucial C/D box snoRNA expression determinant, seems to be less important than previously reported according to most of our predictors, as it occupies the 23rd predictive rank out of 34 features (Fig. 4A). The discrepancy seen between what we describe here and what is assumed in the literature is likely explainable by the fact that our approach encompasses all 1541 human snoRNAs, not only the few extensively studied at the time of the reports (Hirose and Steitz 2001; Richard et al. 2006; Vincenti et al. 2007). Further investigating feature importance but at the single snoRNA level, we also provide, based on the collected features, a convincing explanation to the case of host genes that harbor multiple snoRNAs with varying expression statuses (Fig. 5). Applying our predictors to other species (by first validating their performance on the mouse species), we highlight that most vertebrates express only between 13% and 45% of their annotated snoRNAs and that the proportion of expressed snoRNAs is significantly anticorrelated with the total number of snoRNAs annotated in a species’ genome (Fig. 6).

**Figure 7.**
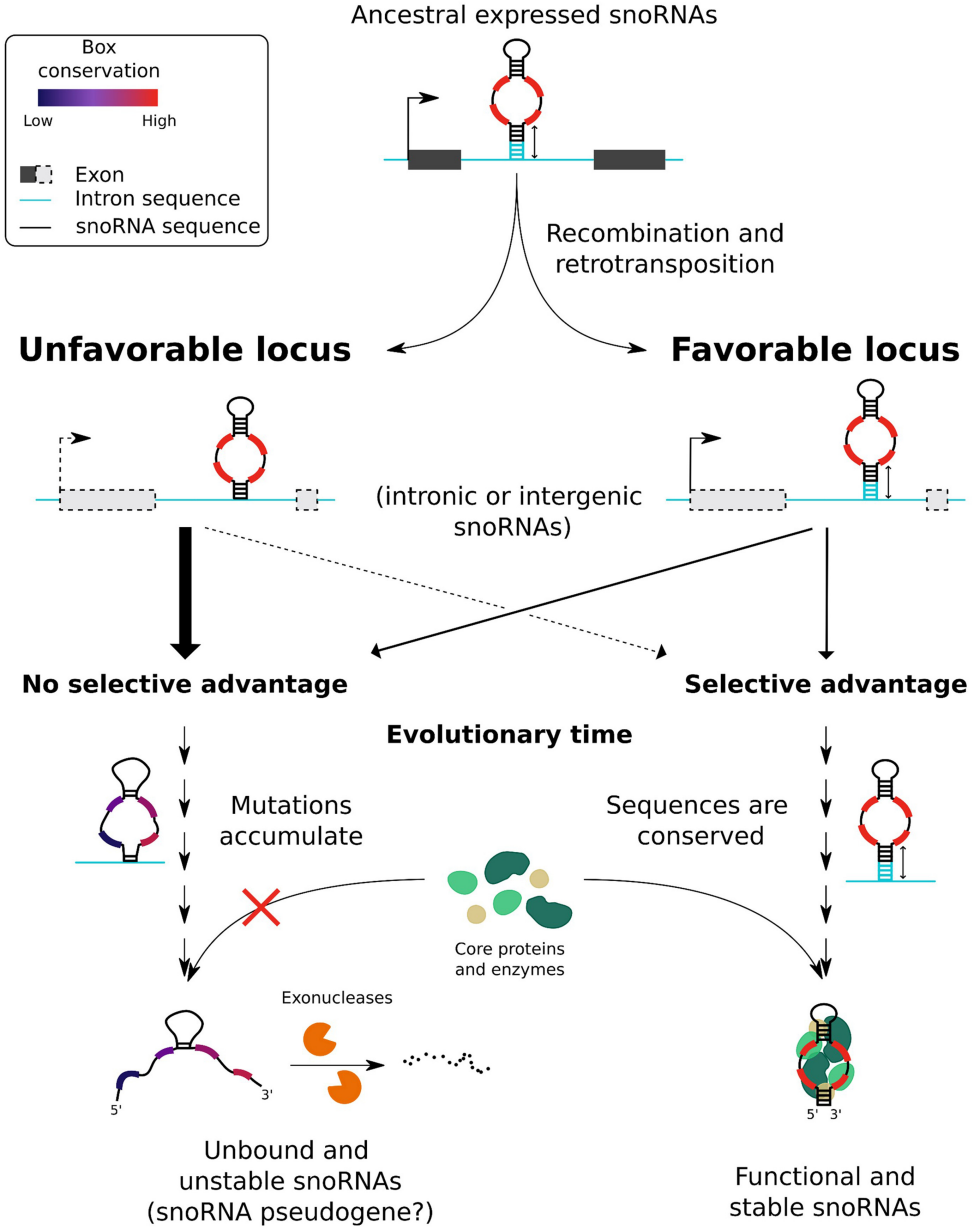
Model explaining the low proportion of expressed snoRNAs annotated in vertebrate genomes. Expressed ancestral snoRNAs presumably had box motifs close to their current consensus sequence, stable terminal stem, and global structure, as well as a genomic context favorable to their expression owing to a nearby promoter. Following a recombination or retrotransposition event, the new locus of these copied snoRNAs might prove to be favorable or unfavorable to their expression, such as containing or not a nearby promoter and flanking sequences likely to promote the formation of a stable terminal stem (the dashed line for the promoter representing a possible lack of promoter). If the newly copied snoRNA induced a gain in fitness, there likely was selective pressure to conserve its sequence and its flanking regions, promoting the binding of core proteins and enzymes to the expressed snoRNA to stabilize its structure and generate a stable and functional snoRNA. Conversely, snoRNAs integrated in unfavorable loci, if transcribed, likely had their binding to core proteins and enzymes hindered by the lack of stable terminal stem, therefore not providing a selective advantage for the organism and thereby allowing mutation accumulation within these snoRNAs. If transcribed, these unstable snoRNAs were then likely degraded by exonucleases and thus represent the nonexpressed snoRNAs (possibly snoRNA pseudogenes) in the present vertebrate genome annotations. Of note, only C/D box snoRNAs are represented in the model, but the same conclusions apply to H/ACA box snoRNAs according to our analyses.

Taken together, our results suggest a model in which, throughout time, snoRNAs spread and evolved across genomes, thereby broadening snoRNA repertoires (Fig. 7). Through retrotransposition and recombination events (Weber 2006; Schmitz et al. 2008; Zhang et al. 2010; Bergeron et al. 2021), ancestral snoRNAs, which likely possessed strong consensus motifs as well as a stable secondary structure and terminal stem (i.e., features that are paramount for their stability and expression), would have been copied in new loci that could be either favorable or unfavorable to their expression (Fig. 7). A favorable locus is defined here as one providing an active promoter (either a host gene promoter or an independent promoter in the case of snoRNAs integrated within introns or intergenic regions, respectively) and adequate sequences flanking the snoRNA such that they enable the formation of a stable terminal stem. We hypothesize that a long and stable terminal stem promoted snoRNA expression as it would have served as a pedestal to present the conserved motifs to core proteins and enzymes composing the snoRNP, away from the rest of the intron. These newly copied snoRNAs might then have either conferred a selective advantage to the organism (e.g., via the modulation of ribosome biogenesis or the development of new snoRNA functions) or not. In the latter case, the snoRNA sequences were likely to degenerate, whereas in the former case, because of the gain in fitness that they induced, these snoRNA sequences were likely conserved through positive selection.

Conversely, ancestral expressed snoRNAs could also have been copied in unfavorable loci (Fig. 7), defined by the lack of an active promoter nearby and/or the lack of flanking sequences enabling the formation of a terminal stem. If these newly copied snoRNAs lacked an active upstream promoter, they were presumably never transcribed (and thus not expressed). However, if they lacked only a stable terminal stem, these snoRNAs were probably transcribed, but the binding of core proteins to the snoRNA was likely hindered, producing an unstable snoRNA. Unless these snoRNAs conferred a selective advantage to the organism (which is plausible, yet highly unlikely), it is reasonable to speculate that in both cases (lack of promoter and/or of a stable terminal stem), there was no selective pressure to conserve these newly copied snoRNAs. This likely led to an accumulation of mutations in the snoRNA sequences and, eventually, to an unstable secondary structure, motif degeneration, and further decreased protein binding to these snoRNAs. These degenerate and unstable snoRNAs, if transcribed, would have been rapidly degraded by exonucleases, thus representing the high proportion of nonexpressed snoRNAs we observe in present species. Of note, one cannot rule out the third option of newly integrated copies that have not yet accumulated any mutation, thereby positioning these snoRNAs at the crossroads between neofunctionalization and pseudogenization depending on the effect of future mutations in their sequence.

snoRNA integration in an optimal locus likely happened less often than in an unfavorable locus, as most annotated vertebrate snoRNAs are not expressed, and most of these are encoded within intergenic regions (Supplemental Table S2; Figs. 1A, 6A), which frequently lack active promoters. Furthermore, the fact that most vertebrates are predicted to have only between 13% and 45% of expressed snoRNAs suggests that only a low number of different snoRNAs need to be expressed to ensure a basal and functional level of rRNA modification (Fig. 6C). The most minimalistic species we covered in this study with regard to snoRNAs is the chicken, which presents as few as 130 snoRNAs predicted to be expressed, forming a core group of snoRNAs that are potentially conserved across species. Of note, the proportion of expressed snoRNAs is inversely related to the total number of annotated snoRNAs across vertebrate genomes (Fig. 6D), indicating that the more snoRNA retrotransposition/recombination events a genome harbors, the less likely these events lead to appropriate expression of all snoRNA copies (with the extreme case of the platypus, which was reported to have more than 40,000 full or truncated snoRNA copies, most being located in suboptimal genomic context) (Schmitz et al. 2008).

Another interesting avenue following snoRNA integration in a locus is the development of new functions after some mutations accumulate. Indeed, it is well known that protein-coding gene duplication serves as an evolutionary playground to give rise to new gene functionalities while keeping a parental copy that ensures the original function (Ohno 1970). The same process potentially applies to snoRNAs, as some mutations might affect snoRNA structure and its interactions with other RNAs and proteins. Doing so, these mutated snoRNAs might acquire new targets and cellular roles such as regulating splicing and pre-mRNA stability, processes in which snoRNAs were observed to be involved in recent years (Bergeron et al. 2020; Bratkovič et al. 2020). Notably, our models predicted as expressed nine snoRNAs with favorable features that were not detected in our TGIRT-seq data sets (Supplemental Fig. S15) but that were present in HumanRef samples, suggesting that they might be expressed in other conditions (e.g., other healthy human tissues, tissues under stress, or affected by diseases, etc.). *SNORD86*, one of these FPs, was shown to regulate its host gene splicing and expression level by adopting two alternative structures (Lykke-Andersen et al. 2018). This snoRNA harbors two mutations within its motifs (A > G at the end of both C and C′ boxes), hinting at the possibility that these mutations (and potentially others across its structure) allowed *SNORD86* to switch more easily between the two alternate structures, therefore creating a new regulatory pathway for the cell. It is thus plausible that among expressed snoRNAs harboring some mutations and even among the eight other FP snoRNAs with favorable features, some of these snoRNAs developed or are in the evolutionary process to develop new functions that are yet to be discovered.

In conclusion, our study raises several fundamental questions regarding current snoRNA annotation practices. Because most annotated snoRNAs are not expressed, should these genes even be considered as actual snoRNAs? Furthermore, to what extent should motif degeneration be tolerated when annotating snoRNA genes? For instance, >14% of nonexpressed C/D box snoRNAs have no identifiable C box, and >80% and 30% of nonexpressed H/ACA box snoRNAs have no identifiable H or ACA box, respectively (Fig. 4B; Supplemental Fig. S11). These results challenge the very definition of what is considered a bona fide snoRNA and call into question the reliability of present annotations. This also indicates that current annotation practices are being too permissive in defining what is a snoRNA gene, and underlines that future work will be needed to at least recalibrate, if not reannotate, eukaryote genomes with regard to snoRNAs based on a refined approach encompassing the main expression determinants identified herein. To facilitate snoRNA studies by the community, we propose that the degenerate and nonexpressed snoRNAs be identified as snoRNA pseudogenes in further annotation releases, as they display features that are incompatible with their expression. As snoRNA feature distributions are not always as clear-cut as one would expect between expression statuses (e.g., some nonexpressed snoRNAs harbor highly conserved motifs, whereas some expressed snoRNAs display an unstable terminal stem), it seems that vertebrate genomes are in constant evolution, oscillating between defining snoRNA copies as mere remnants of snoRNA duplication or as building blocks of a future layer of gene expression regulation.

## Methods

### TGIRT-seq data acquisition, processing, and label definition

TGIRT-seq data analysis was performed using our previously described pipeline (Fafard-Couture et al. 2021) on seven biological triplicates of healthy human tissues (breast, ovary, prostate, testis, skeletal muscle, brain, and liver) with our custom human genome annotation file (gene transfer format [GTF]) available at https://zenodo.org/record/6799536/files/hg38_Ensembl_V101_Scottlab_2020.gtf and is described in further details in the Supplemental Methods. An abundance table containing each tissue triplicate sample (given in TPM) was obtained as the output of the pipeline. From this abundance table, 1541 human snoRNAs were extracted (based on the gene biotype “snoRNA” from our custom GTF file). The expression status of each snoRNA was defined as follows: A given snoRNA was considered as expressed if its abundance was >1 TPM in at least one average tissue (average of biological triplicates) and considered as not expressed otherwise.

Mouse abundance data sets were generated by processing six publicly available TGIRT-seq samples (three untreated mESC samples and three treated with retinoic acid) (McCann et al. 2020) through the same data analysis pipeline but using the mouse genome and GTF obtained from Ensembl (version 105, GRCm39 assembly, no supplemental annotations). From the resulting abundance table, snoRNA expression status (expressed or not) was defined as previously described for human snoRNAs.

### Human snoRNA categorical feature extraction

snoRNA type (C/D or H/ACA), target (rRNA, snRNA, or orphan), host gene biotype (protein-coding, noncoding, or intergenic), function (ribosomal protein, ribosome biogenesis and translation, RNA binding processing, splicing, other, poorly characterized, functional noncoding RNA, nonfunctional noncoding RNA, or intergenic), susceptibility to NMD, and propensity to harbor a dual-initiation promoter were obtained from snoDB (version 1.0) (Bouchard-Bourelle et al. 2020) and as previously described (Fafard-Couture et al. 2021). The expression status for host genes (host is expressed, host is not expressed, or intergenic) was defined, using the same procedure described above for human snoRNA label definition, but this time applied to host genes.

### Human snoRNA numerical feature calculation

snoRNA length (i.e., the number of nucleotides) was derived directly from our custom GTF file. Box score was determined using custom Python scripts based on Hamming distance (snoRNAs with box motifs closer to their consensus sequences getting lower box score and vice versa). For C/D box snoRNAs, C and D boxes were identified in snoRNA sequences by prioritizing exact consensus match (RUGAUGA and CUGA, respectively, where R is a purine) over motifs with mismatches compared with the consensus (up to three and two mismatches were allowed, respectively; no motif was returned otherwise). As snoRNA length varies between snoRNA of the same type (Supplemental Fig. S2E), the search for C and D motifs was confined, respectively, within the first and last 20 nt of snoRNA sequences. As C′ and D′ boxes are often degenerate (Henras et al. 2004), an alternative approach was used to identify these boxes: The best C′/D′ pair was chosen based on the fact that it should minimize the total C′/D′ Hamming distance (i.e., the sum of C′ and D′ Hamming distances based on their respective RUGAUGA and CUGA consensus motifs). These motifs were searched for between the 21st and 21st-to-last nucleotide of the snoRNA sequence (with the found D′ box being always upstream of the found C′ box). The final box score was then obtained by summing up the C, D, and C′/D′ Hamming distances, ranging from zero (representing a C/D box snoRNA with perfect C, D, C′, and D′ boxes) to a theoretical 22 (representing a C/D box with completely degenerate C, D, C′, and D′ boxes). For H/ACA box snoRNAs, as H (ANANNA, where N is any nucleotide) and ACA motifs are relatively short and simple, no mismatches were allowed when searching for these motifs in snoRNA sequences. The H motif was searched for in unpaired (hinge) regions; the ACA motif, in the last 10 nt of the sequences. Applying the same Hamming distance strategy, the final box score was obtained by summing up H and ACA Hamming distances, ranging from zero (representing an H/ACA box snoRNA with perfect H and ACA boxes) to nine (representing an H/ACA box snoRNA with completely degenerate H and ACA boxes).

For intronic snoRNAs, total intron number per host gene, intron length in which the snoRNA is encoded, absolute intron rank (counting in which intron the snoRNA is encoded from the 5′ or 3′ end), relative intron rank (counting in which intron the snoRNA is encoded from the 3′ end divided by the total number of introns in the host gene), and snoRNA distance to the upstream and downstream exons were retrieved from our custom GTF file. Branchpoint location in introns of intronic snoRNAs was predicted using branchpointer (version 1.16.0) with default parameters (Signal et al. 2018). snoRNA distance to the branchpoint was then retrieved by computing the distance between the snoRNA 3′ end and the best-predicted branchpoint (the one with the highest probability). snoRNA global stability (given in minimal free energy [MFE]) was computed using RNAfold with default parameters from the version 2.4.14 of the Vienna RNA package (Lorenz et al. 2011). snoRNA terminal stem stability was computed by first collecting the flanking 15 nt upstream of and downstream from each mature snoRNA to consider the genomic sequence surrounding the snoRNA and its intronic context (Deschamps-Francoeur et al. 2014). For C/D box snoRNAs, these sequences were then both extended by 5 nt (internal snoRNA nucleotides). For H/ACA box snoRNAs, we hypothesized that a potential terminal stem could also be formed as the 5′ and 3′ ends of H/ACA box snoRNAs are often closely located in snoRNA structural representation (Supplemental Fig. S1; Kalvari et al. 2018). The H/ACA box flanking sequences were thus extended by five internal nucleotides and only three internal nucleotides from the 5′ and 3′ ends, respectively, because we suspected that the ACA motif (which is often located 3 nt upstream of the snoRNA 3′ end) might not participate in the terminal stem nucleotide pairing. For each snoRNA, a terminal stem stability (in MFE) was computed from the pairing of the two extended flanking regions using RNAcofold with default parameters from the version 2.4.14 of the Vienna RNA package (Lorenz et al. 2011). Finally, a terminal stem length score was defined for each snoRNA based on the previously identified terminal stems. This score was calculated as the number of intermolecular paired nucleotides between the two extended flanking regions minus the number of nucleotides within gap(s) inside the stem (a low and high score representing approximately a small and long stem, respectively).

### Processing of eCLIP and PAR-CLIP data sets

The eCLIP data sets of AQR and dyskerin (DKC1) were obtained from the ENCODE Consortium (Van Nostrand et al. 2020). The PAR-CLIP data sets of fibrillarin, NOP56, and NOP58 were generated and obtained from a previous study (Kishore et al. 2013). Their analysis is described in the Supplemental Methods.

### Collection of mouse snoRNA features

Mouse snoRNA global stability, terminal stem stability, and box score were computed as described earlier with human snoRNAs. As terminal stem stability and box score needed snoRNA type information to be computed, this information was retrieved from RNAcentral for most of the mouse snoRNAs (RNAcentral Consortium 2021). For intronic snoRNAs, host genes were defined as such if they overlapped with a snoRNA on the same strand using the mouse GTF file previously described. Host gene biotype information was also retrieved from the mouse GTF file. As the mouse TGIRT-seq data mentioned earlier only included small RNAs, host gene abundance was obtained from another study comprising biological duplicate samples of 13 adult mouse tissues (bone marrow, cerebellum, cerebral cortex, heart, kidney, liver, lung, spleen, intestine, olfactory bulb, placenta, testis, and thymus), as well as biological duplicate samples of six embryonic tissues (mESC, brain, heart, liver, limb, and fibroblasts) (Shen et al. 2012). An abundance table (in TPM) containing all of these samples was obtained from recount3 using the Monorail analysis pipeline with default parameters (Wilks et al. 2021). The expression status for mouse host genes was defined the same way as with human host genes. The final mouse input features were thus composed of snoRNA global stability, terminal stem stability, box score, and host gene expression status. Based on these four features, redundant snoRNAs (i.e., those with exactly the same four feature values) were filtered out to limit positive or negative bias when computing the predictor accuracy on the mouse data set.

### Collection of snoRNA features across other vertebrate species

snoRNA features were collected for the chimpanzee (*P. troglodytes*, Pan_tro_3.0), gorilla (*Gorilla gorilla*, gorGor4), macaque (*Macaca mulatta*, Mmul_10), rabbit (*Oryctolagus cuniculus*, OryCun2.0), rat (*Rattus norvegicus*, mRatBN7.2), cow (*Bos taurus*, ARS-UCD1.2), platypus (*Ornithorhynchus anatinus*, mOrnAna1.p.v1), chicken (*G. gallus*, GRCg6a), western clawed frog (*Xenopus tropicalis*, Xenopus_tropicalis_v9.1), and zebrafish (*D. rerio*, GRCz11) based on their respective Ensembl GTF file (version 105). Species snoRNA global stability, terminal stem stability, box score, host gene definition, and host gene expression status were defined as described above for mouse snoRNAs, except for the fact that the abundance data sets (in TPM) were obtained directly from the Bgee expression database (Bastian et al. 2021). The proportion of snoRNAs predicted to be expressed or not, depending on the snoRNA type or the host gene biotype, is shown per species in the Supplemental Table S2.

### Input feature processing and spliting into tuning, training, and test data sets

For human snoRNAs, all categorical and numerical features were merged into a single table (Supplemental Table S1). Categorical features were then one-hot-encoded into separate features, and redundant features were removed (e.g., the “intergenic” feature was kept only once, although it was generated every time a feature related to host gene was one-hot-encoded). The same procedure was applied to mouse and vertebrate species snoRNAs. For human snoRNAs only, the resulting input feature table was shuffled and split into three sets in a stratified way (i.e., keeping the same ratio of expressed vs. not expressed labels in each set): the tuning, training, and test sets (respectively, 10%, 80%, and 10% of all human snoRNAs) (Fig. 3A). This process was performed in parallel 10 different times, ensuring that all the snoRNAs were partitioned in one of the 10 test sets (each test set having its unique set of snoRNAs) (Supplemental Fig. S5). Then, the remainder of snoRNAs was split between the tuning and validation sets, allowing the prediction of the expression status of all snoRNAs one time across the 10 iterations. Feature scaling was then applied in each separate set using standardization. For mouse and other vertebrate species snoRNAs, features were scaled using the mean and variance of the human snoRNA training set selected iteration.

### Hyperparameter tuning, training, and testing of models

Five types of predictive models were tuned, trained, and tested in this study: logistic regression, support vector machine, *k*-nearest neighbors, random forest, and gradient boosting. For human snoRNAs, a total of 50 different models were optimized (five models across 10 iterations) for each combination of input features that was tested (all input features, only box score, top three or top four features). Hyperparameter tuning was performed for each model using the grid search algorithm on their respective tuning set and search space (with a stratified threefold cross-validation strategy). Using their respective tuned hyperparameters, the models were then trained on their respective training set to optimize their parameters. Prediction accuracy of these trained models was then assessed through their prediction of the expression status of human snoRNAs present in their respective test set. The final predicted value (and its corresponding value in the confusion matrix, i.e., TP, TN, FP, or FN) was defined based on an ensemble approach, meaning that it was chosen based on the most common prediction across the three selected models (logistic regression, support vector machine, and random forest).

### Model prediction on the mouse and other vertebrate species data sets

To determine whether the predictive models could be applied to species other than human such as the mouse, 25 new models were optimized (the same five model types across five new iterations). The tuning and training sets were composed of, respectively, 10% and 90% of the human snoRNAs, which was followed by a stratified nested fivefold cross-validation approach, whereas the test set was composed of all the filtered mouse snoRNAs. Hyperparameter tuning, training, and testing was performed as described earlier with human snoRNAs. Because of its high prediction accuracy and stability across the five iterations, the logistic regression model (iteration with random state [seed] of 42 to split the data sets) was chosen to predict the expression status of snoRNAs in the 10 vertebrate species mentioned above. All of these steps were implemented using the version 0.23.2 of the scikit-learn library (Pedregosa et al. 2011).

### Model interpretability via SHAP values

Model interpretability was assessed using the SHAP package (version 0.39.0) (Lundberg and Lee 2017). For each model, a SHAP value was computed for all features across the human snoRNAs present in their respective test set. The mean of SHAP value (SHAP values being in absolute value) distribution per feature was computed, and the predictive rank per feature was defined based on these means: the highest mean corresponding to the most predictive rank (i.e., rank 1) and the lowest being the least predictive rank.

### Visualization and statistical analyses

Graphs were generated using either pandas (version 1.2.0), Matplotlib (version 3.3.4), seaborn (version 0.11.1), logomaker (version 0.8) (Tareen and Kinney 2020), SHAP (version 0.39.0) (Lundberg and Lee 2017), and exported images from the Integrative Genomics Viewer (IGV; version 2.4.18) (Robinson et al. 2011). Statistical analyses were performed with SciPy (version 1.5.2); statistical significance was defined at (*) *P* < 0.05, (**) *P* < 0.01, and (***) *P* < 0.001.

### Software availability

All code is available within a reproducible Snakemake workflow (version 6.0.5) (Köster and Rahmann 2012) that is available at GitHub (https://github.com/etiennefc/Abundance_determinants_snoRNA) and as Supplemental Code.

## Supplementary Material

Supplemental Material
