# Supplementary material for: Motif conservation, stability, and host gene expression are the main drivers of snoRNA expression across vertebrates
Source: Genome Res. 2023 Apr;33(4):525–40. doi: 10.1101/gr.277483.122 (PMC10234308; doi:10.1101/gr.277483.122)
Supplement: Supplemental Material [file supp_33_4_525__DC1.html]

Motif conservation, stability, and host gene expression are the main drivers of snoRNA expression across vertebrates — Motif conservation, stability, and host gene expression are the main drivers of snoRNA expression across vertebrates — Supplemental Material 

# Motif conservation, stability, and host gene expression are the main drivers of snoRNA expression across vertebrates

## Supplemental Material

- Supplemental\_Methods.pdf
- Supplemental\_Figures\_S1\_S18\_Table\_S2.pdf
- Supplemental\_Table\_S1.xlsx
- Supplemental\_code\_repository.zip
