# Supplementary material for: Motif conservation, stability, and host gene expression are the main drivers of snoRNA expression across vertebrates
Source: Genome Res. 2023 Apr;33(4):525–40. doi: 10.1101/gr.277483.122 (PMC10234308; doi:10.1101/gr.277483.122)
Supplement: Supplemental Material [file supp_gr.277483.122_Supplemental_Methods.pdf]

Supplemental Methods (Fafard-Couture *et al.* 2023)

TGIRT-seq data acquisition, processing, and label definition

TGIRT-seq data analysis was performed using our previously described pipeline (Fafard-Couture et al. 2021) on seven biological triplicates of healthy human tissues (breast, ovary, prostate, testis, skeletal muscle, brain and liver) with our custom human genome annotation file available at https://zenodo.org/record/6799536/files/hg38_Ensembl_V101_Scottlab_2020.gtf and CoCo, a tool designed for the quantification of embedded genes such as snoRNAs (Deschamps-Francoeur et al. 2019). This annotation was built upon the version 101 of the Ensembl gene transfer format (GTF) file (based on the GRCh38.p13 human genome (version 101)) that was supplemented with snoRNAs from the human snoRNA database snoDB (version 1.0) (Bouchard-Bourelle et al. 2020). An abundance table containing each tissue triplicate samples (given in TPM) was obtained as the output of the pipeline. From this abundance table, 1541 human snoRNAs were extracted (based on the gene biotype “snoRNA” from our custom GTF file). The expression status of each snoRNA was defined as follows: a given snoRNA was considered as expressed if its abundance was greater than 1 TPM in at least one average tissue (average of biological triplicates), and considered as not expressed otherwise. In addition, publicly available HumanRef samples (three replicates sequenced by TGIRT-seq) (Nottingham et al. 2016) were also processed through the same bioinformatic pipeline and used in the analysis of FP snoRNAs (therefore not considered when defining the snoRNA expression status). These data are available through the Sequence Read Archive at https://www.ncbi.nlm.nih.gov/sra/ and can be accessed with the accession number SRX1426160. The remaining TGIRT-seq data analysed during the current study are available in the Gene Expression Omnibus repository at https://www.ncbi.nlm.nih.gov/geo/ and can be accessed with the accession numbers GSE126797, GSE157846 and GSE140623.

GTEx host gene abundance data processing

To verify the impact of changing the source of transcriptomic data on the ‘host gene expression’ feature, abundance data (in TPM) from matched GTEx tissues (biological triplicates of the same seven healthy

human tissues mentioned above but from the GTEx project) and unmatched GTEx tissues (biological triplicates of healthy human adrenal gland, colon, spleen, heart, kidney, thyroid and nerve) were downloaded from the GTEx project (version 8) (Lonsdale et al. 2013). The GTEx-based host gene expression status was then defined as described above.

Analysis of eCLIP and PAR-CLIP datasets

The eCLIP datasets of AQR and dyskerin (DKC1) were obtained from the ENCODE consortium (Van Nostrand et al. 2020). The chosen AQR datasets were generated in the HepG2 and K562 human cell lines, whereas the chosen DKC1 dataset was generated in the HepG2 cell line. The PAR-CLIP datasets of fibrillarin, NOP56 and NOP58 were generated and obtained from a previous study in the HEK293 human cell line (Kishore et al. 2013). For each RNA-binding protein, overlapping peaks with at most 1 nucleotide distance (within a same sample or between replicates) were merged as single peaks. Peaks of all RNA-binding proteins except AQR were filtered to keep only those with an overlap of at least half the length of the snoRNA. A final filtering step was applied to keep only peaks with a $p < 0.01$ for the eCLIP datasets and those with a score greater than 10 in PAR-CLIP datasets.

Statistical analyses

Sensitivity, specificity and simple matching coefficient (SMC) were calculated with the following equations:

$$Sensitivity = \frac{True\ positives}{True\ positives + False\ negatives}$$

$$Specificity = \frac{True\ negatives}{True\ negatives + False\ positives}$$

$$SMC = \frac{Number\ of\ matching\ attributes}{Sum\ of\ all\ attributes}$$

where the number of matching attributes corresponds to the number of expressed and non-expressed host genes that are defined as such based on both the TGIRT-seq and GTEx datasets, and where the sum of all

attributes corresponds to the total number of host genes (regardless of if their expression status was defined the same way or differently based on the TGIRT-seq and GTEx datasets).

Supplemental Methods references

Bouchard-Bourelle P, Desjardins-Henri C, Mathurin-St-Pierre D, Deschamps-Francoeur G, Fafard-Couture É, Garant J-M, Elela SA, Scott MS. 2020. snoDB: an interactive database of human snoRNA sequences, abundance and interactions. *Nucleic Acids Research* **48**: D220–D225. doi:10.1093/nar/gkz884.

Deschamps-Francoeur G, Boivin V, Abou Elela S, Scott MS. 2019. CoCo: RNA-seq read assignment correction for nested genes and multimapped reads (B Berger, Ed.). *Bioinformatics* **35**: 5039–5047. doi:10.1093/bioinformatics/btz433.

Fafard-Couture É, Bergeron D, Couture S, Abou-Elela S, Scott MS. 2021. Annotation of snoRNA abundance across human tissues reveals complex snoRNA-host gene relationships. *Genome Biology* **22**:. doi:10.1186/s13059-021-02391-2.

Kishore S, Gruber AR, Jedlinski DJ, Syed AP, Jorjani H, Zavolan M. 2013. Insights into snoRNA biogenesis and processing from PAR-CLIP of snoRNA core proteins and small RNA sequencing. *Genome Biology* **14**: R45. doi:10.1186/gb-2013-14-5-r45.

Lonsdale J, Thomas J, Salvatore M, Phillips R, Lo E, Shad S, Hasz R, Walters G, Garcia F, Young N, et al. 2013. The Genotype-Tissue Expression (GTEx) project. *Nature Genetics* **45**: 580–585. doi:10.1038/ng.2653.

Van Nostrand EL, Freese P, Pratt GA, Wang X, Wei X, Xiao R, Blue SM, Chen JY, Cody NAL, Dominguez D, et al. 2020. A large-scale binding and functional map of human RNA-binding proteins. *Nature 2020 583:7818* **583**: 711–719. doi:10.1038/s41586-020-2077-3.

Nottingham RM, Wu DC, Qin Y, Yao J, Hunicke-Smith S, Lambowitz AM. 2016. RNA-seq of human

reference RNA samples using a thermostable group II intron reverse transcriptase. *RNA (New York, NY)* **22**: 597–613. doi:10.1261/rna.055558.115.
