# Supplementary material for: Motif conservation, stability, and host gene expression are the main drivers of snoRNA expression across vertebrates
Source: Genome Res. 2023 Apr;33(4):525–40. doi: 10.1101/gr.277483.122 (PMC10234308; doi:10.1101/gr.277483.122)
Supplement: Supplemental Material [file supp_gr.277483.122_Supplemental_Figures_S1_S18_Table_S2.pdf]

Supplemental figures S1-S18 and table S2
(Fafard-Couture et al., 2023)

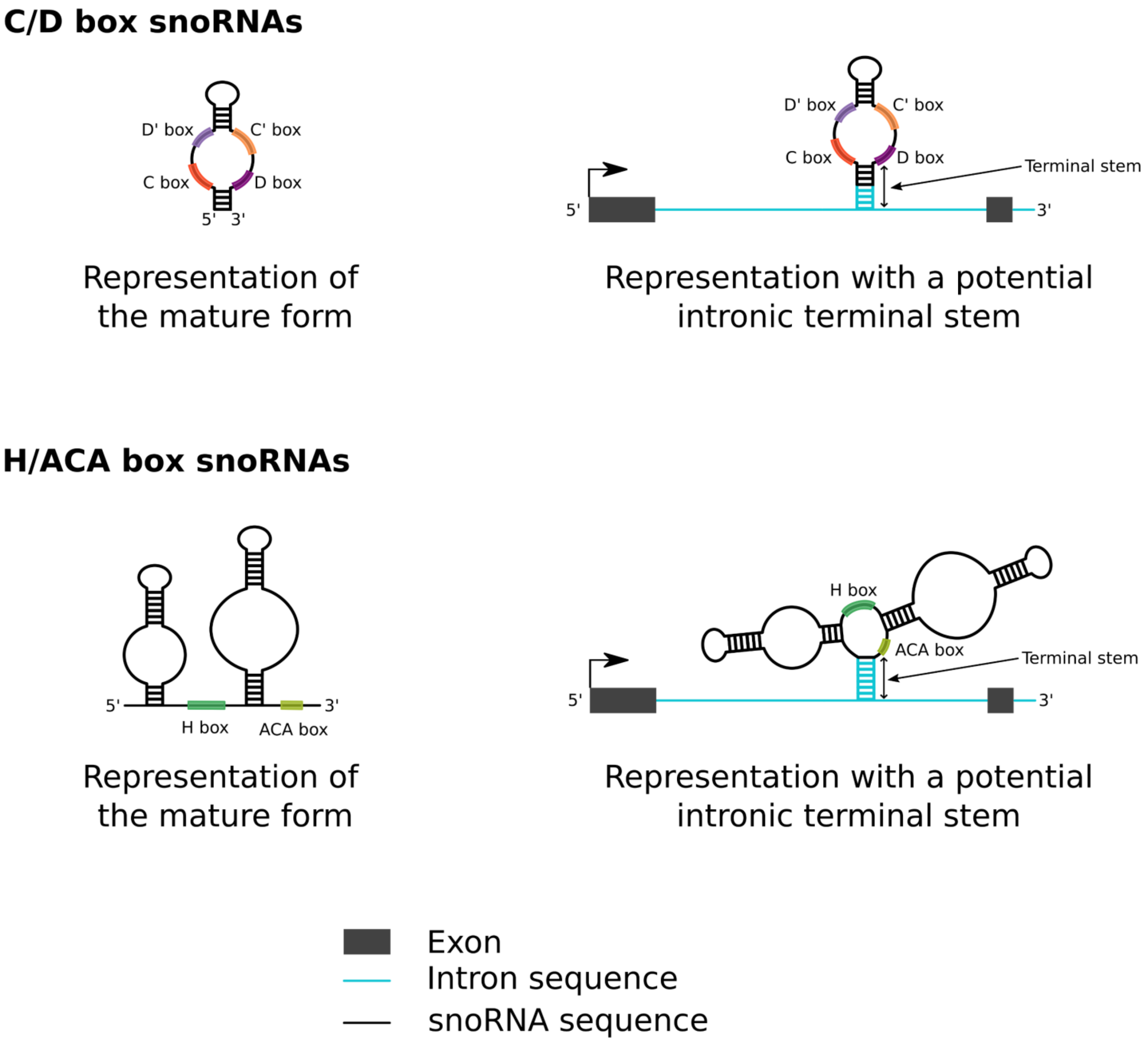

Figure S1 (Supplementary to Figure 1). SnoRNA structure representations. Both C/D and H/ACA box snoRNAs are represented as their mature form (left panel) or as their potential structure within their transcribed locus of origin, with the formation of an intronic terminal stem (right panel).

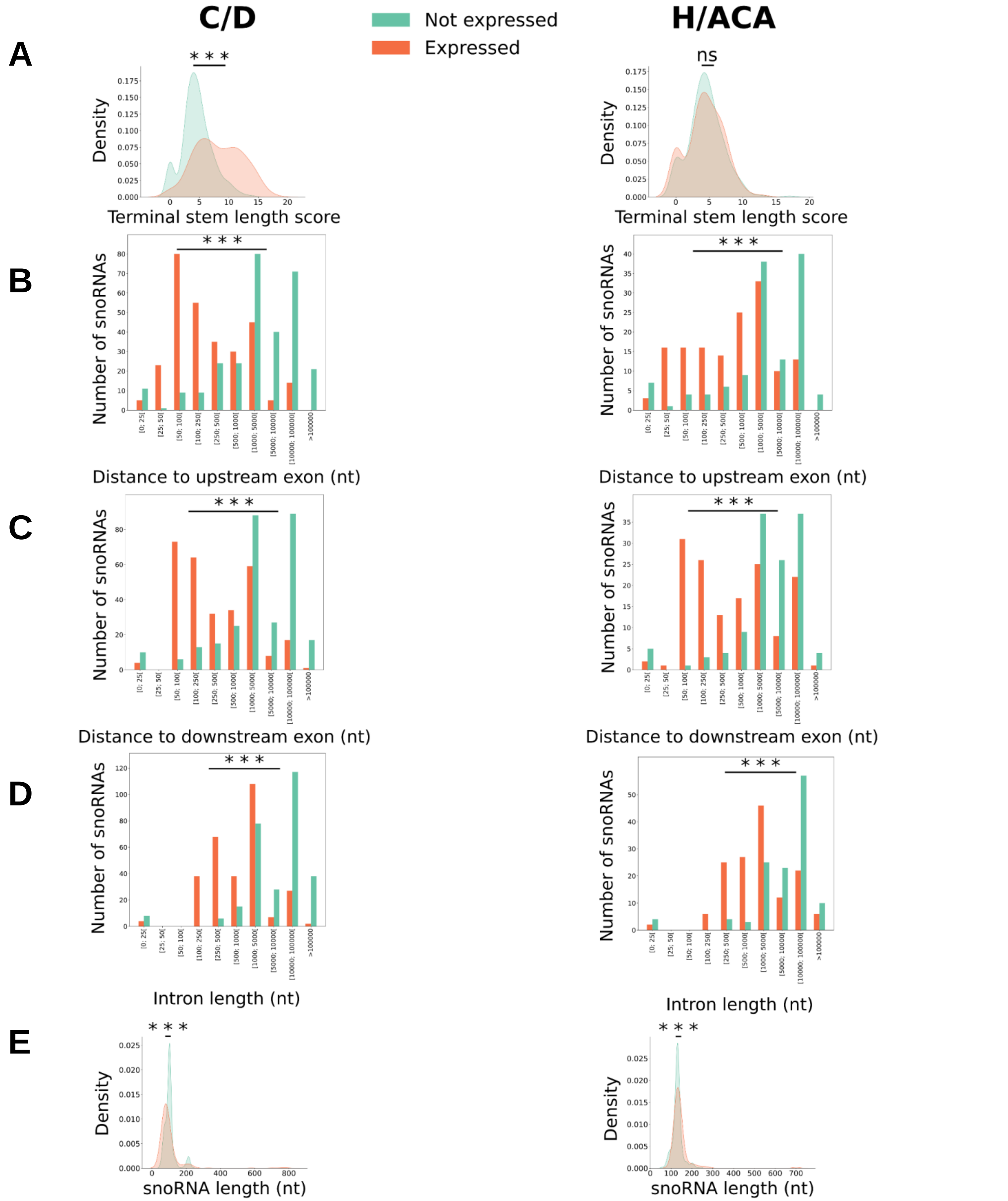

Figure S2 (Supplementary to Figure 1). Distribution of numerical features for C/D and H/ACA box snoRNAs according to their expression status. (**A-E**) Distribution of C/D (left panel) and H/ACA box (right panel) snoRNAs features such as their terminal stem length score (the C/D box snoRNA distributions are significantly different at $***p < 2 \times 10^{-47}$, Mann-Whitney *U* test), (**A**), their distance to the upstream (**B**) and downstream (**C**) exon within their host gene, their intron length (the distributions are significantly different at $***p < 7 \times 10^{-86}$ and $***p < 2 \times 10^{-37}$ for C/D and H/ACA box snoRNAs respectively, Mann-Whitney *U* test) (**D**) and snoRNA length (**E**), depending on their expression status (ns: not significant).

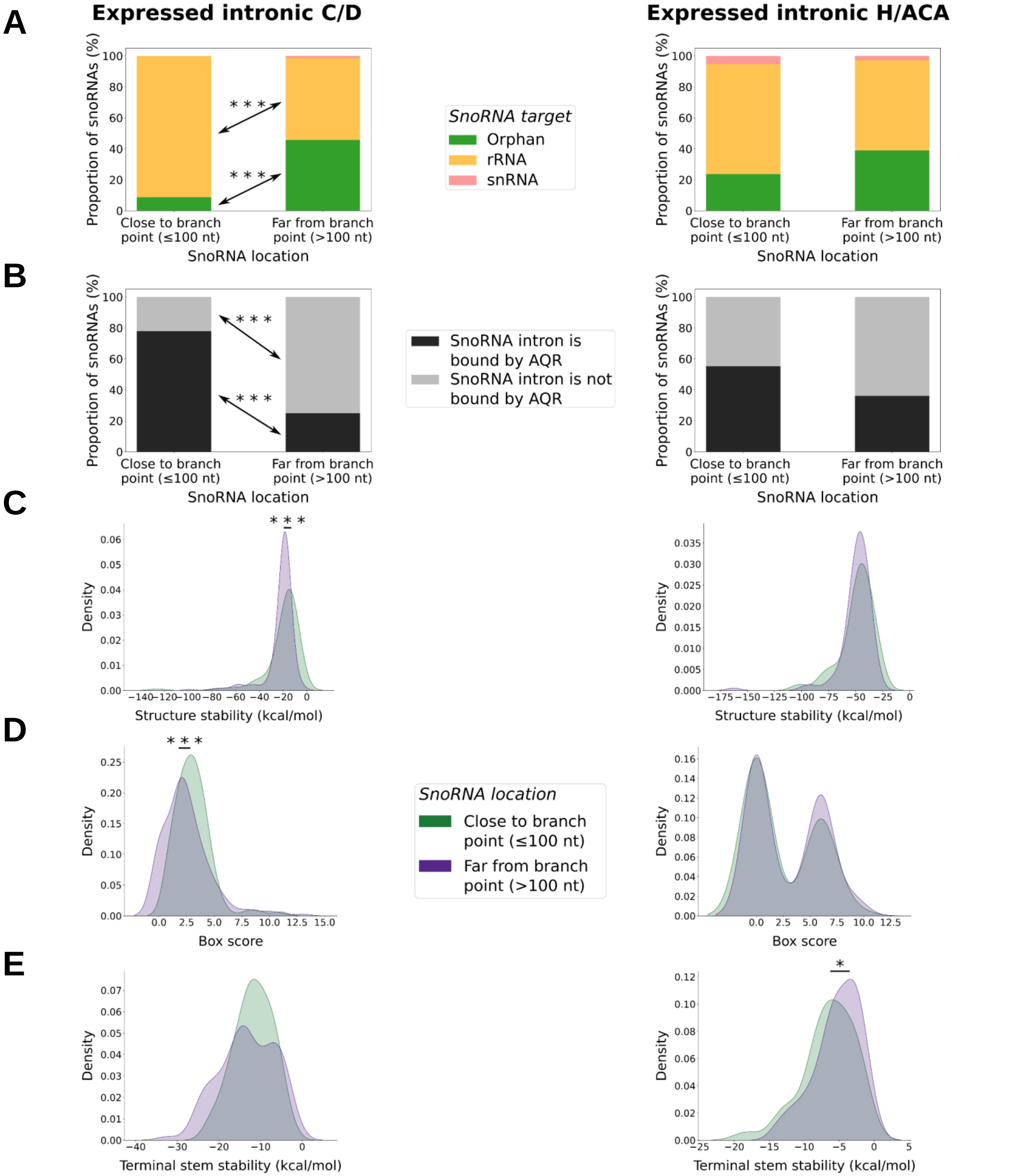

Figure S3 (Supplementary to Figure 1). Distribution of characteristics for expressed intronic C/D and H/ACA box snoRNAs according to their location with regards to the branch point. (**A-E**) Distribution of C/D (left panel) and H/ACA box (right panel) snoRNAs characteristics such as their target (***$p < 7 \times 10^{-11}$, Fisher's exact test) (**A**), the binding of Aquarius (AQR) in their intron (***$p < 2 \times 10^{-17}$, Fisher's exact test) (**B**), their whole structure stability (the C/D box snoRNA distributions are significantly different at ***$p < 6 \times 10^{-5}$, Mann-Whitney *U* test) (**C**), their box score (the C/D box snoRNA distributions are significantly different at ***$p < 5 \times 10^{-4}$, Mann-Whitney *U* test) (**D**) and their terminal stem stability (the H/ACA box snoRNA distributions are significantly different at *$p < 0.05$, Mann-Whitney *U* test) (**E**) .

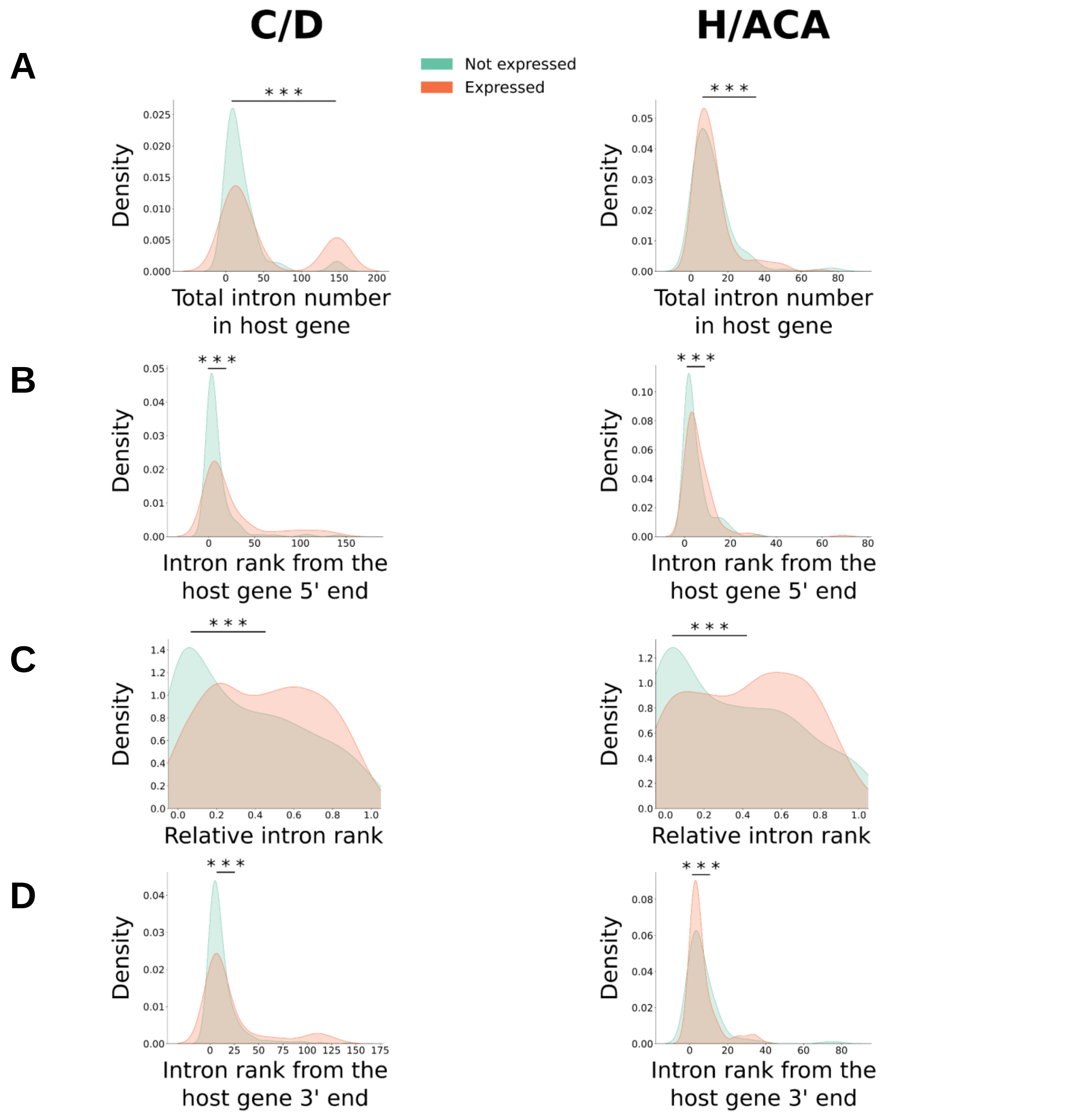

Figure S4 (Supplementary to Figure 1). Distribution of intronic features for C/D and H/ACA box snoRNAs according to their expression status. (**A-D**) Distribution of C/D (left panel) and H/ACA box (right panel) intronic snoRNAs features such as the number of introns in the host gene in which they are encoded (the distributions are significantly different at $***p < 0.001$, Mann-Whitney *U* test) (**A**), their intron rank from the host gene 5' end (the distributions are significantly different at $***p < 9 \times 10^{-24}$ and $***p < 5 \times 10^{-17}$ for C/D and H/ACA box snoRNAs respectively, Mann-Whitney *U* test) (**B**), their relative intron rank (for clarity purpose in this figure, the relative rank is represented by counting from the 5' end of the host gene, i.e. the intron in which the snoRNA is encoded divided by the total number of introns in the host gene) (the distributions are significantly different at $***p < 2 \times 10^{-32}$ and $***p < 3 \times 10^{-18}$ for C/D and H/ACA box snoRNAs respectively, Mann-Whitney *U* test) (**C**) and their intron rank from the host gene 3' end (the distributions are significantly different at $***p < 2 \times 10^{-32}$ and $***p < 3 \times 10^{-22}$ for C/D and H/ACA box snoRNAs respectively, Mann-Whitney *U* test) (**D**), depending on their expression status.

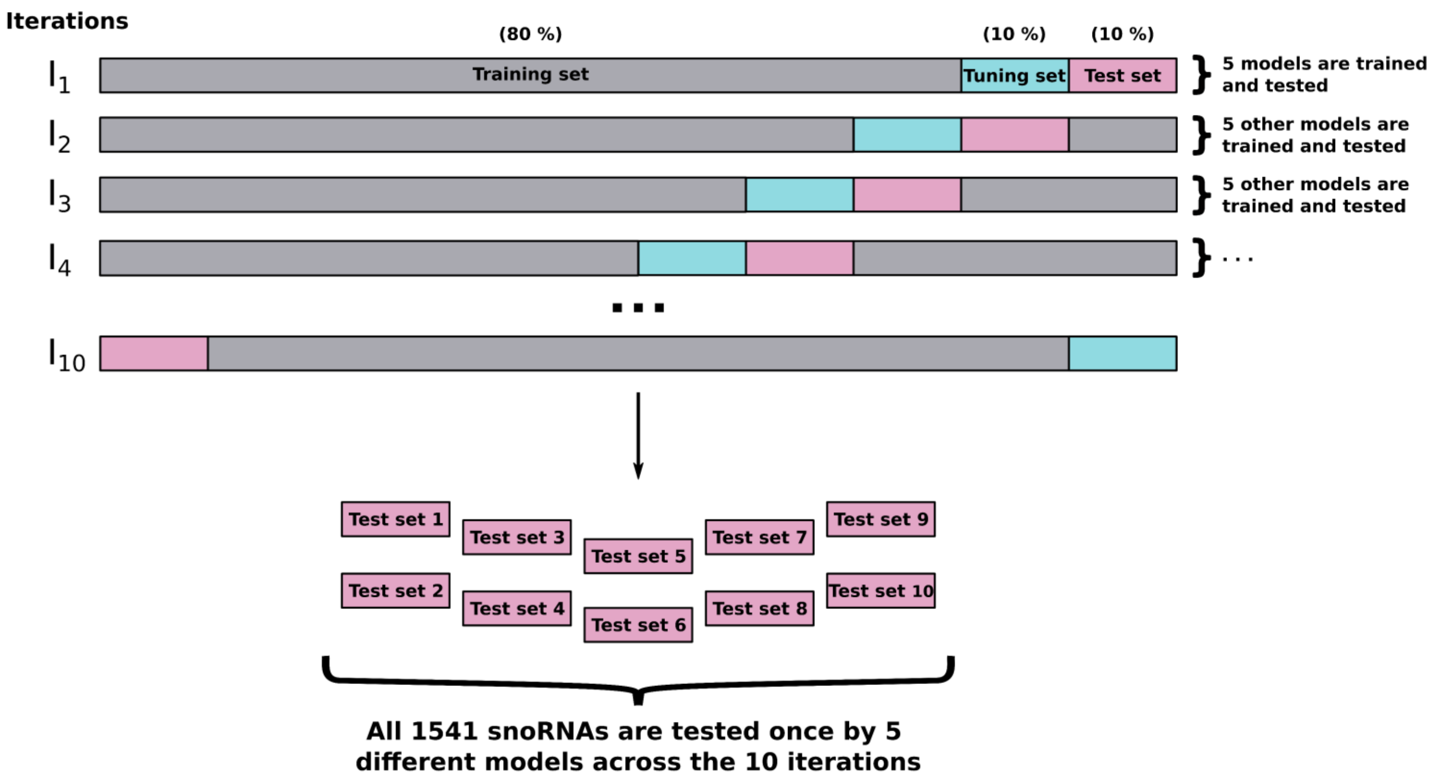

Figure S5 (Supplementary to Figure 3). SnoRNAs were split in training, tuning and test sets across ten different iterations to predict the expression status of all 1541 snoRNAs once across the iterations. For each iteration, a 10 % of snoRNAs was used to tune the hyperparameters of the 5 types of models, a 80 % of snoRNAs was used to train the models and a 10 % that is different across all iterations was used to test the models. Doing so, 5 models were trained per iteration. By aggregating all of the results of the 10 test sets together, the expression status of each snoRNA is thereby predicted once across all the iterations.

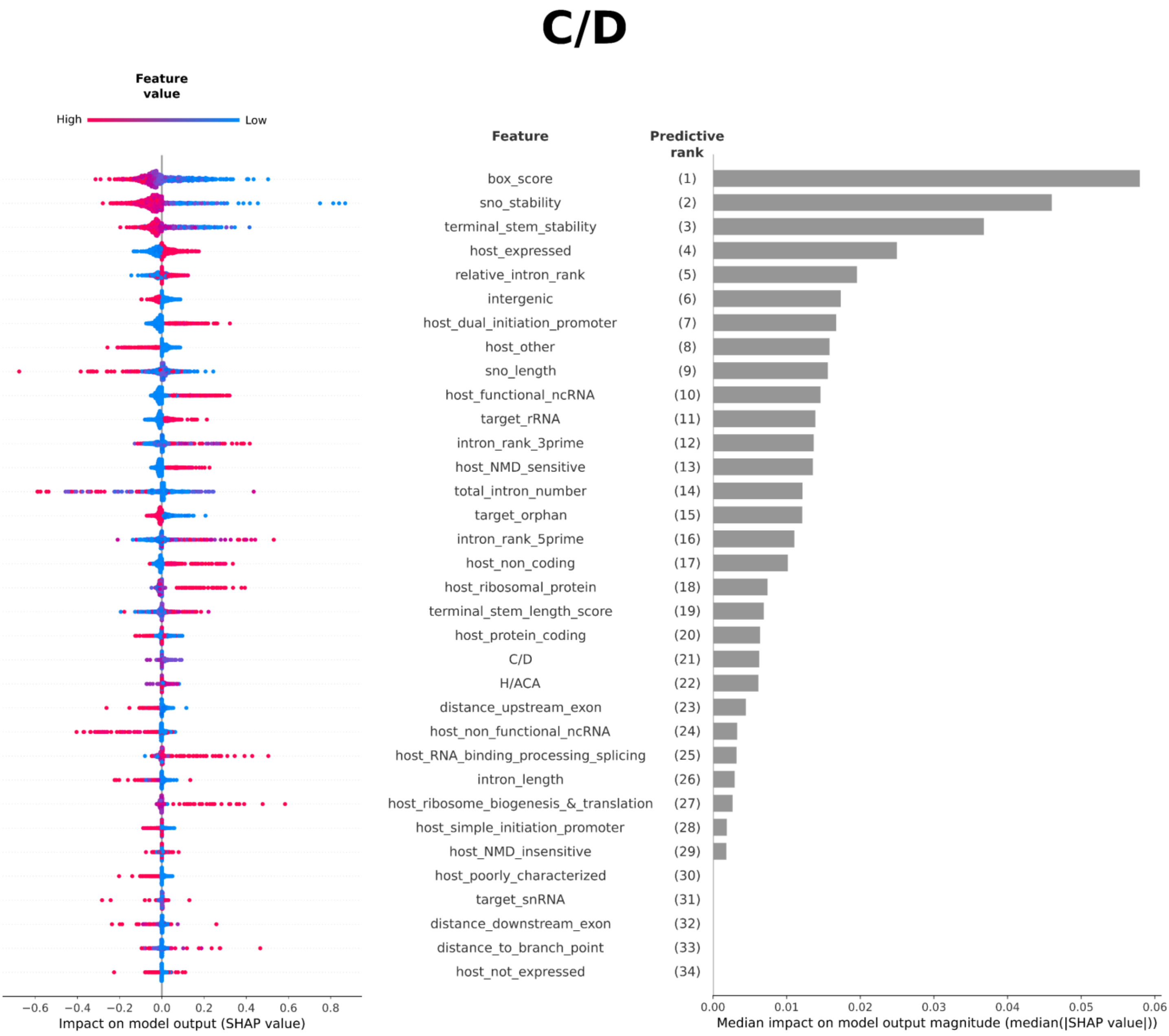

Figure S6 (Supplementary to Figure 4). Different box types rely on the same features to be expressed; box motif close to the consensus sequence and high stability of the global structure or of the terminal stem are associated with positive prediction (snoRNAs being predicted to be expressed). (Left panel) SHAP summary plots displaying features ordered by predictive rank from top to bottom (based on the median of each distribution of absolute value of SHAP values) for C/D box snoRNA classification across iterations based on the Support Vector Machine classifier. The impact on the model for each feature (either positive or negative impact which influences the prediction to be respectively “expressed” or “not expressed”) is represented by the SHAP values on the x axis. Each dot corresponds to one snoRNA present in one of the 10 test set iterations. The dots are colored with regards to their feature value (high and low values being represented respectively in red and blue). (Right panel) Bar chart showing for each feature the median of the distribution of absolute value of SHAP values. The values correspond to the median impact of each feature on the prediction made by the Support Vector Machine classifier on C/D box snoRNAs.

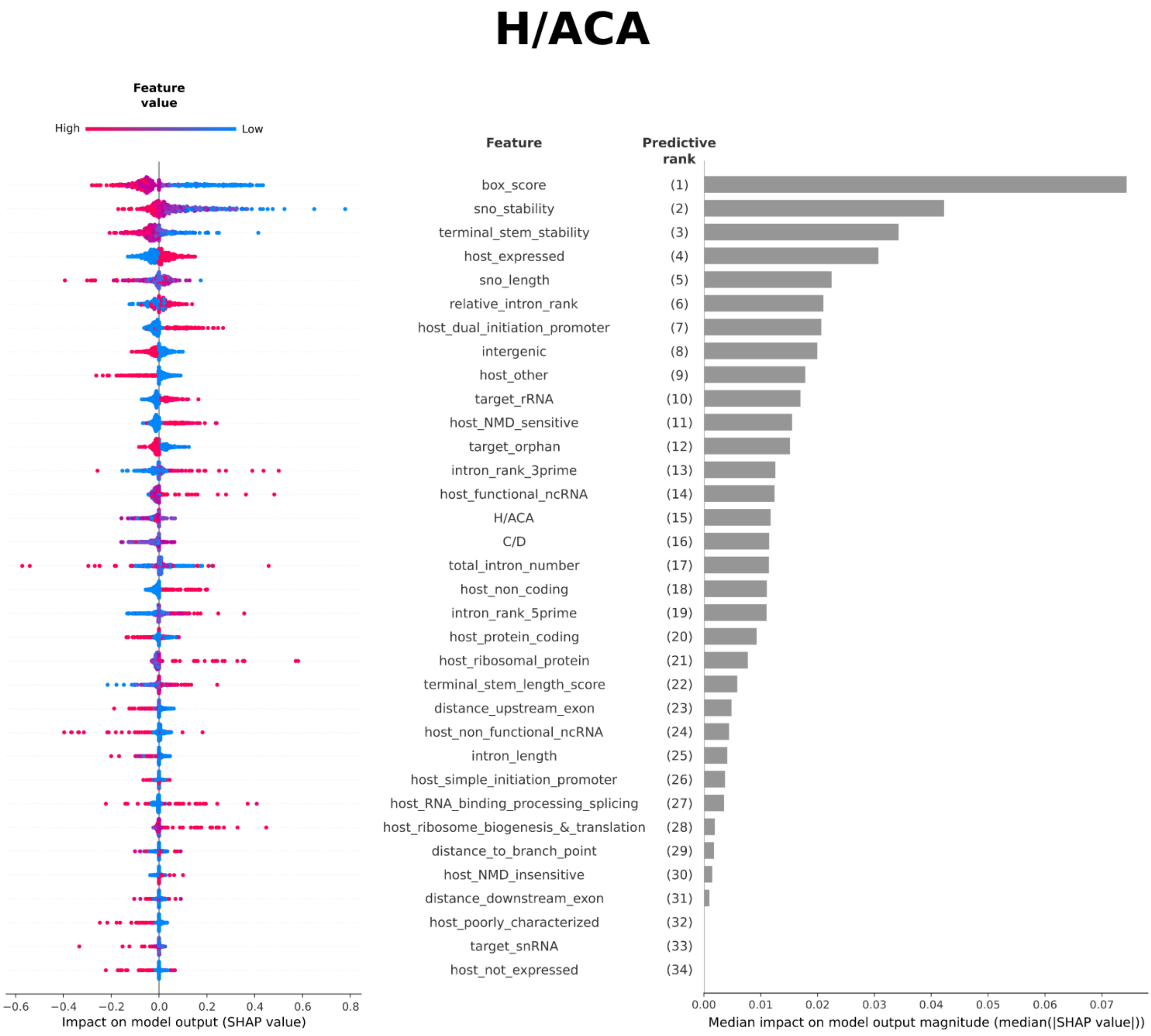

Figure S7 (Supplementary to Figure 4). Different box types rely on the same features to be expressed; box motif close to the consensus sequence and high stability of the global structure or of the terminal stem are associated with positive prediction (snoRNAs being predicted to be expressed). (Left panel) SHAP summary plots displaying features ordered by predictive rank from top to bottom (based on the median of each distribution of absolute value of SHAP values) for H/ACA box snoRNA classification across iterations based on the Support Vector Machine classifier. The impact on the model for each feature (either positive or negative impact which influences the prediction to be respectively “expressed” or “not expressed”) is represented by the SHAP values on the x axis. Each dot corresponds to one snoRNA present in one of the 10 test set iterations. The dots are colored with regards to their feature value (high and low values being represented respectively in red and blue). (Right panel) Bar chart showing for each feature the median of the distribution of absolute value of SHAP values. The values correspond to the median impact of each feature on the prediction made by the Support Vector Machine classifier on H/ACA box snoRNAs.

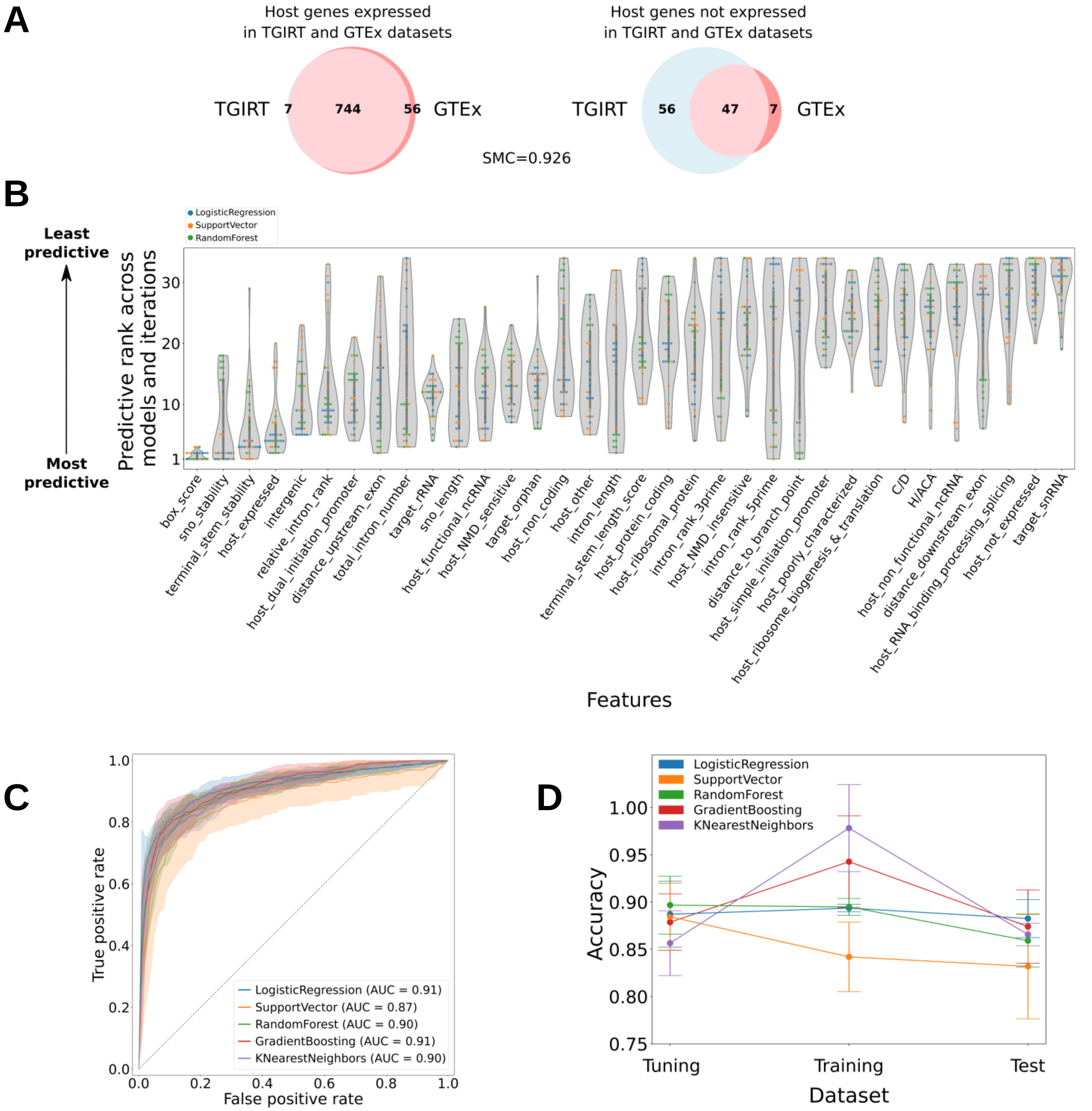

Figure S8 (Supplementary to Figures 3, 4 and 6). Models trained with all features but using host gene expression status based on the matched GTEx datasets show high predicting performance similar to models trained based on TGIRT-Seq datasets. (**A**) Venn diagrams showing the intersection between snoRNA host genes considered as expressed (left panel) or not expressed (right panel) based on the TGIRT-Seq datasets or the matched-tissues GTEx datasets. The concordance between the two datasets is shown by the Simple Matching Coefficient (SMC). (**B**) Violin plots showing the predictive rank of each input features across all selected models and iterations (using matched GTEx expression status). (**C**) ROC curves showing the average true and false positive rates of all models on the test dataset across the 10 iterations based on the features shown in (**B**). The colored areas above and below each curve represent ± 1 standard deviation for each classifier. The average AUC is shown for the five classifiers. (**D**) Scatter plots showing the average accuracies (± standard deviation) of all models on the tuning, training and test datasets across the 10 iterations based on the features shown in (**B**).

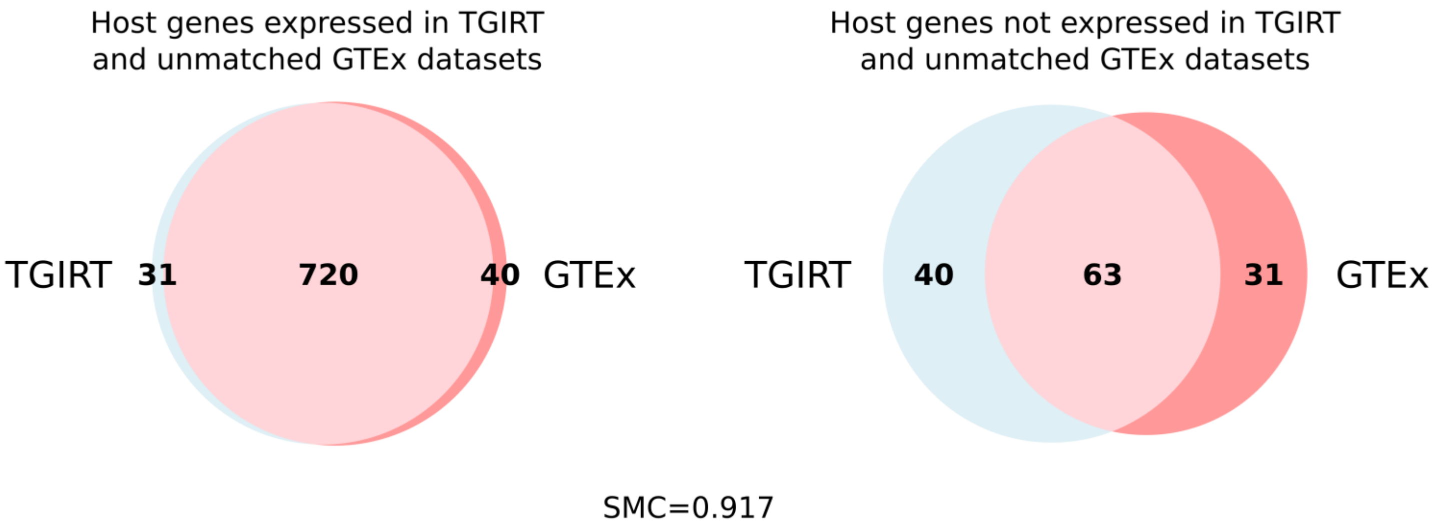

Figure S9 (Supplementary to Figures 3, 4 and 6). The host gene expression status feature is highly concordant whether it is derived from TGIRT-Seq datasets or from unmatched tissue GTEx datasets. Venn diagrams showing the intersection between snoRNA host genes considered as expressed (left panel) or not expressed (right panel) based on the TGIRT-Seq datasets or the unmatched GTEx datasets. These datasets (unlike the GTEx datasets shown in the previous figure S8) are triplicate samples from the unmatched tissues (i.e. not found in the TGIRT-Seq datasets) that are the adrenal gland, colon, spleen, heart, kidney, thyroid and nerve tissues. The concordance between the two datasets is shown by the Simple Matching Coefficient (SMC).

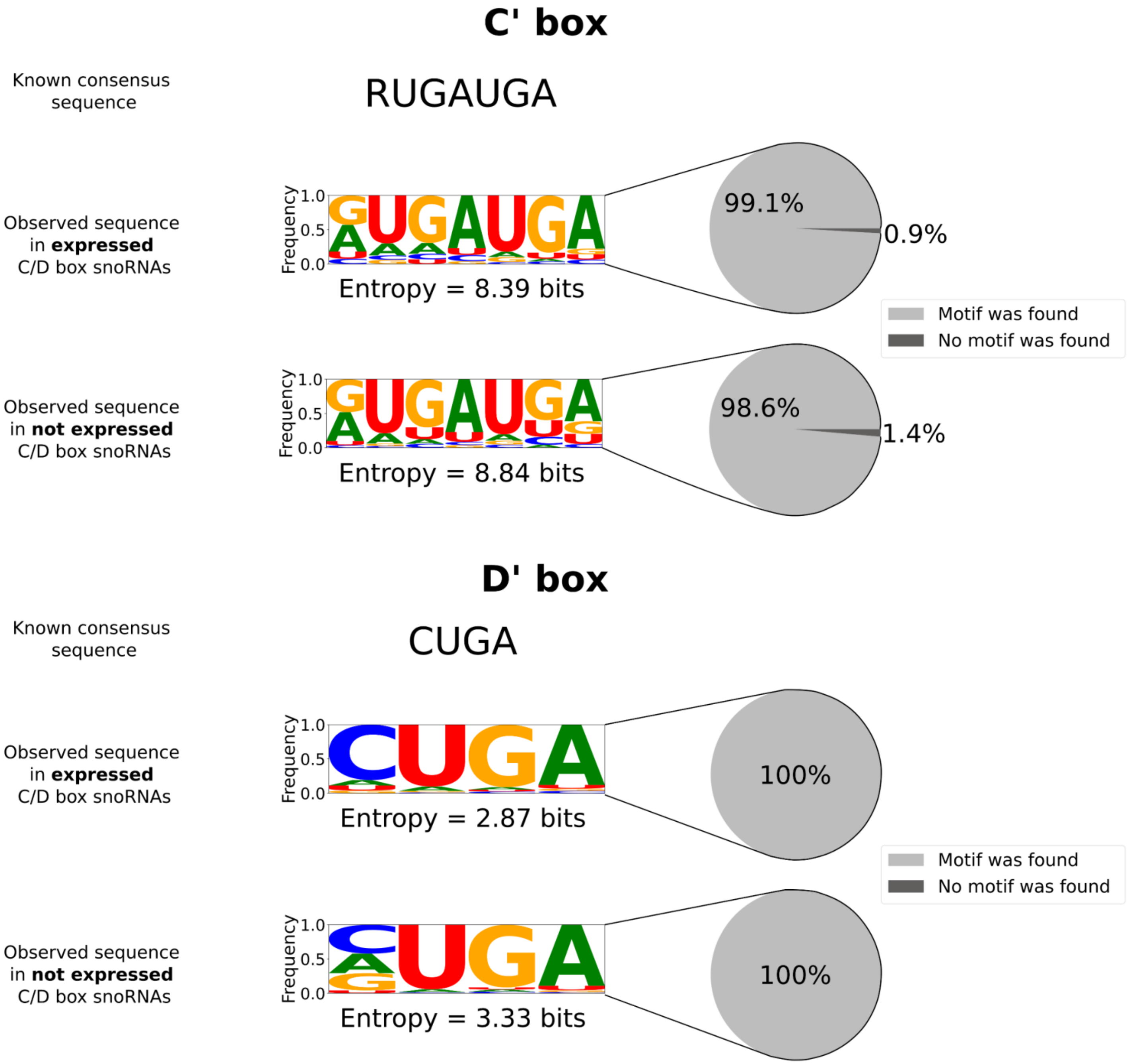

Figure S10 (Supplementary to Figure 4). **Expressed C/D box snoRNAs display motifs that are more similar to the known consensus than not expressed snoRNAs.** (Left panel) Logos showing the frequency of observed C’ (upper panel) and D’ (lower panel) box motifs in expressed C/D box snoRNAs compared to not expressed C/D box snoRNAs. The logos are generated only from snoRNAs in which a motif could be found. The R in the C’ box known consensus sequence corresponds to any purine (A or G). Cumulative Shannon entropy (sum of the entropy per nucleotide) was computed for each logo. (Right panel) Pie charts representing the proportion of C/D box snoRNAs in which a motif could be found or not.

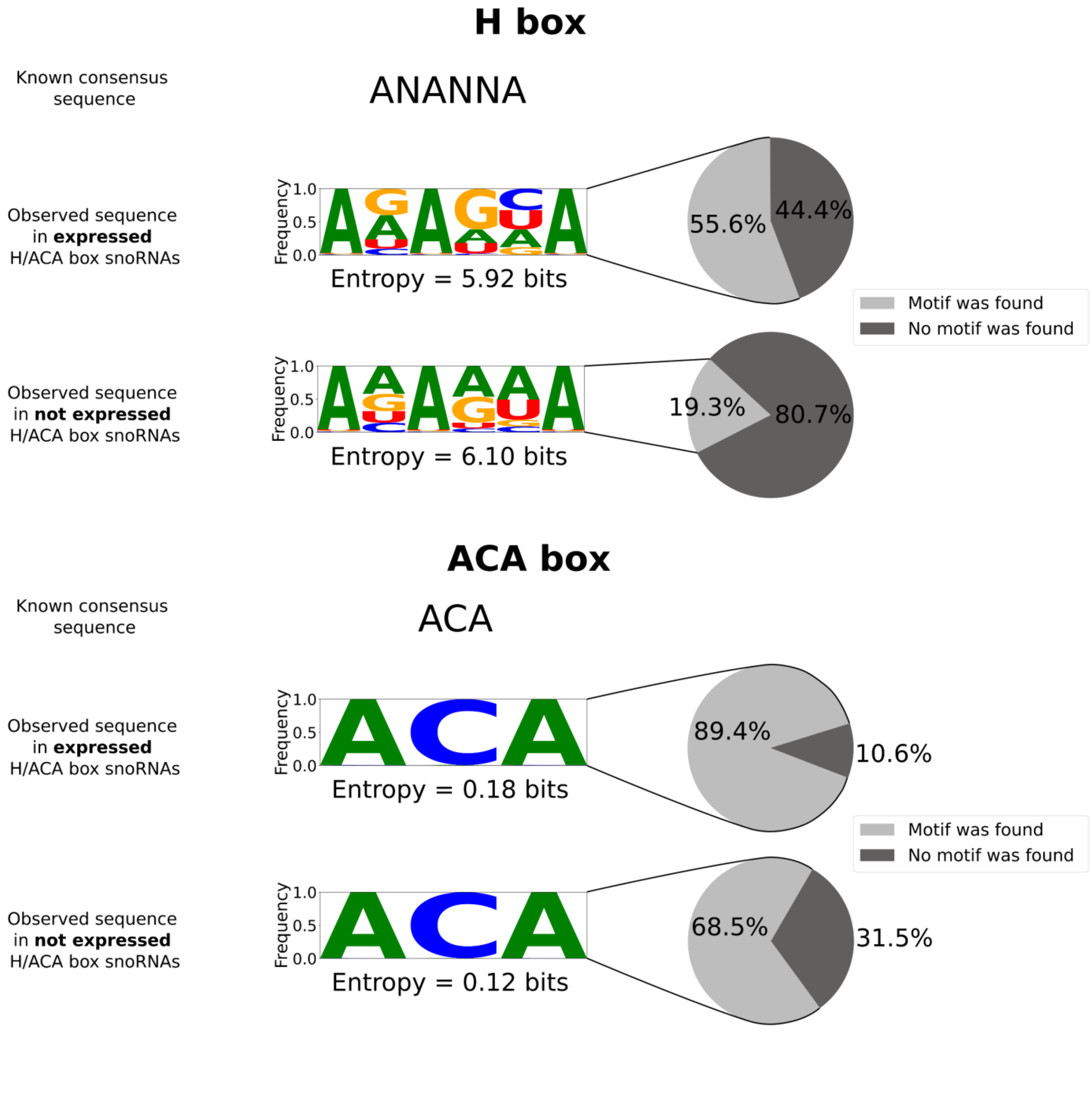

Figure S11 (Supplementary to Figure 4). **Expressed H/ACA box snoRNAs display motifs that are more similar to the known consensus than not expressed snoRNAs.** (Left panel) Logos showing the frequency of observed H (upper) and ACA (lower) box motifs in expressed H/ACA box snoRNAs compared to not expressed H/ACA box snoRNAs. The N in the H box known consensus sequence correspond to any nucleotide (A, U, C or G). Cumulative Shannon entropy (sum of the entropy per nucleotide) was computed for each logo. (Right panel) Pie charts representing the proportion of H/ACA box snoRNAs in which a motif could be found or not.

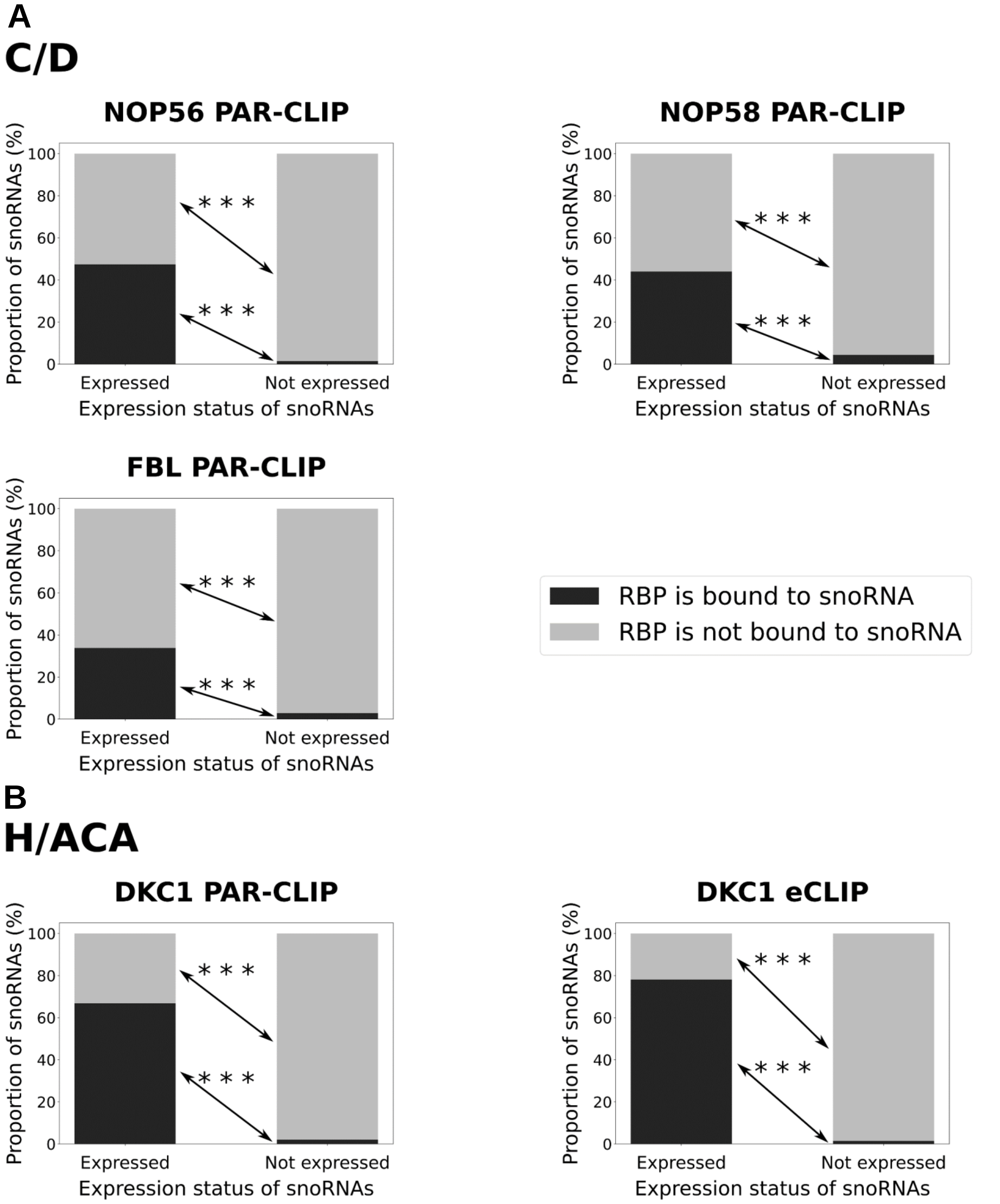

Figure S12 (Supplementary to Figure 4). Expressed snoRNAs show enriched binding of core proteins. (**A**) Proportion of expressed and non-expressed C/D box snoRNAs bound by NOP56, NOP58 and fibrillarin (FBL) based on PAR-CLIP datasets (***p < 3 × 10^{-41}, Fisher's exact test). (**B**) Proportion of expressed and non-expressed H/ACA box snoRNAs bound by dyskerin (DKC1) based on PAR-CLIP and eCLIP datasets (***p < 2 × 10^{-58}, Fisher's exact test).

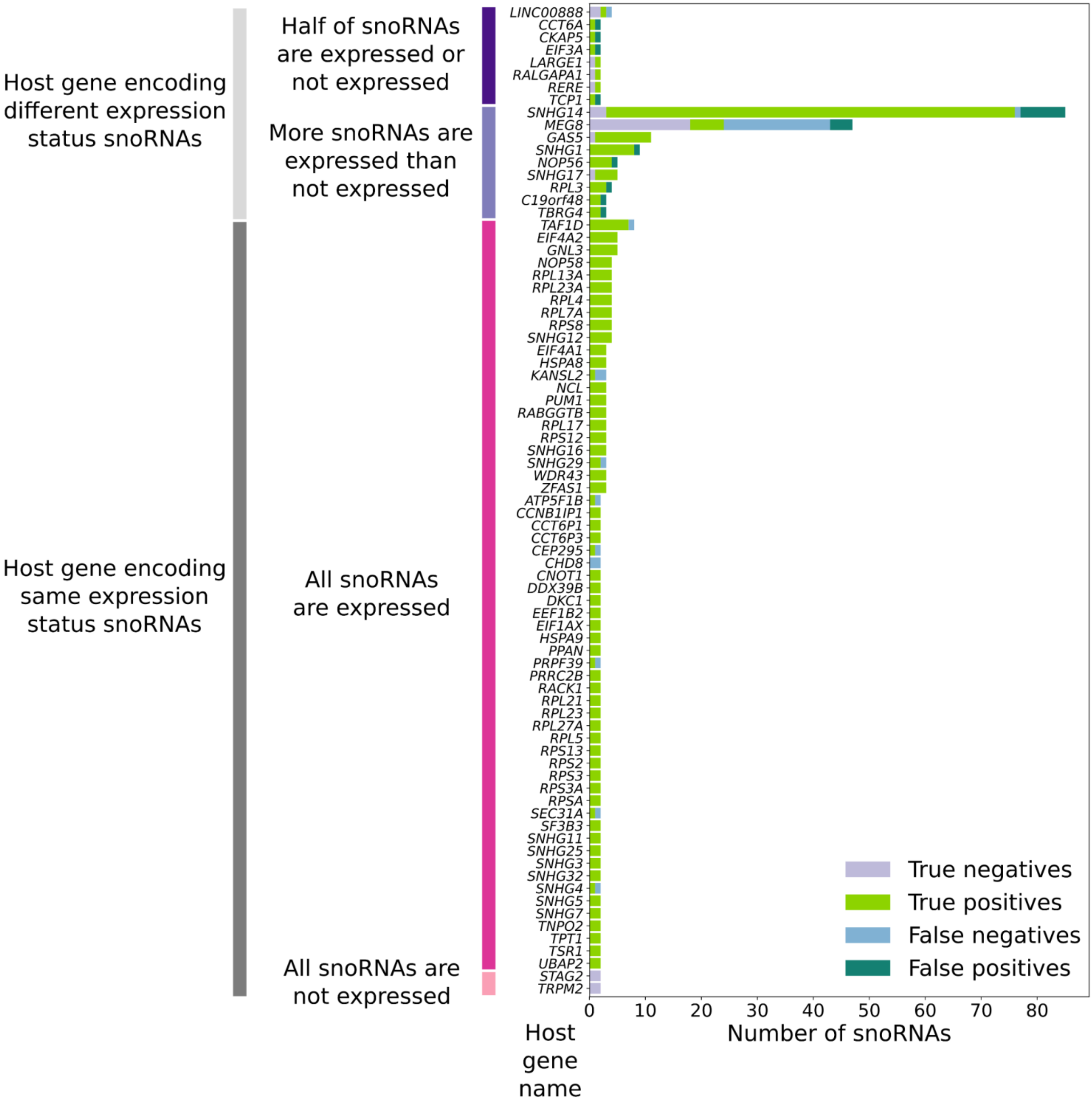

Figure S13 (Supplementary to Figure 5). SnoRNAs encoded within the same host gene are often accurately predicted and are also often only two within the same host gene. Bar chart showing the number of snoRNAs predicted as true negatives/positives and false negatives/positives per host gene encoding multiple snoRNAs. The bars are grouped based on whether the host gene encodes snoRNAs with different or same labels and also based on the expression category of the snoRNAs within the same host gene (half of snoRNAs are expressed or not expressed; more snoRNAs are expressed than not expressed; all snoRNAs are expressed; all snoRNAs are not expressed). Each subgroup of host genes-snoRNAs are then ordered by descending number of snoRNAs per host gene.

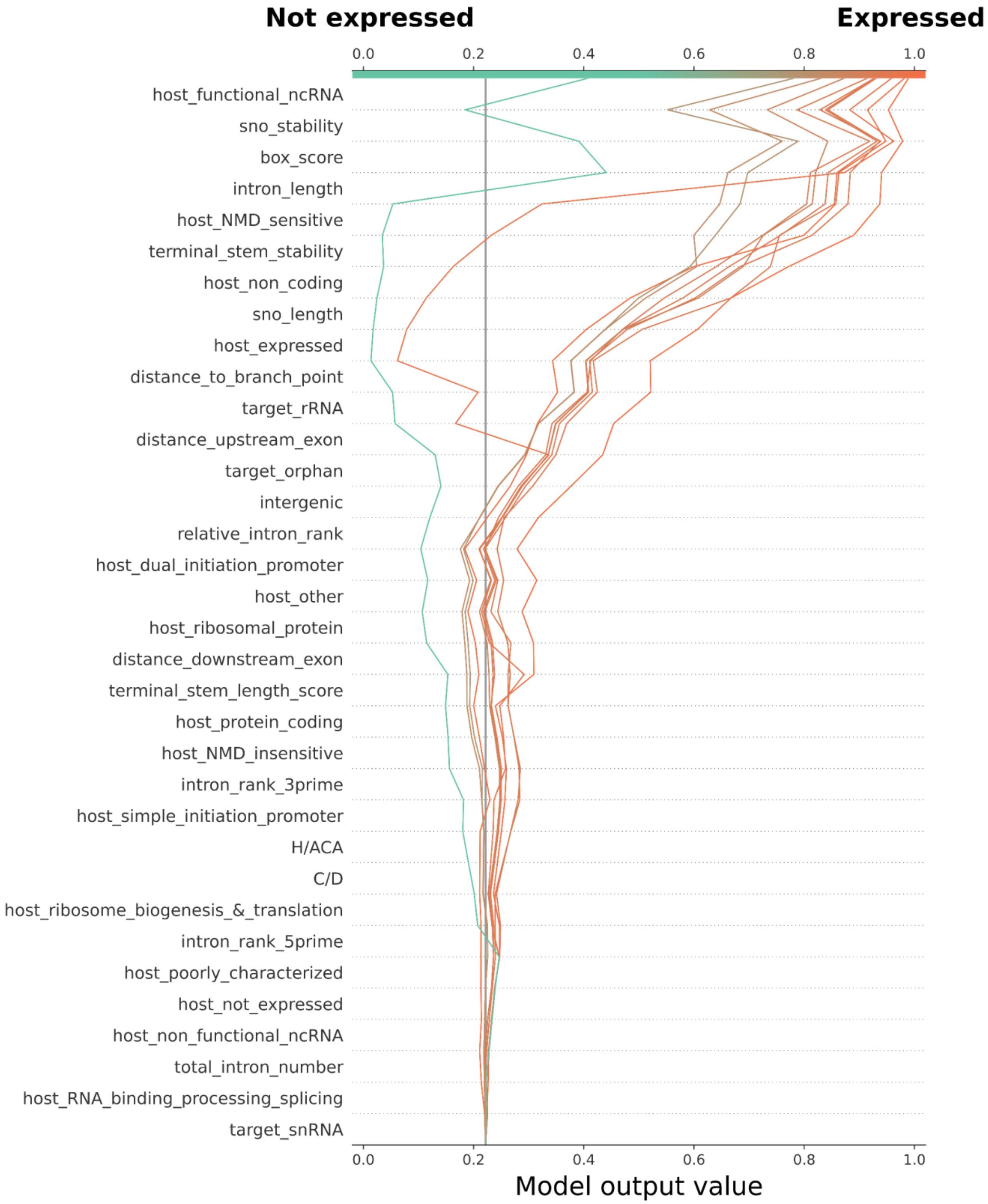

Figure S14 (Supplementary to Figure 5). The Logistic Regression model also predicts accurately the expression status of all snoRNAs in the *GAS5* host gene. Decision plot showing the ''decision process'' of the Logistic Regression classifier in predicting if *GAS5* snoRNAs are expressed or not. From bottom to top, the decision starts at the base value of ~0.25 (average of the classifier output over the training set) and ends at the model output value (between 0 i.e. ''Not expressed'' and 1 i.e. ''Expressed''). This decision process is influenced positively (towards the ''Expressed'' output) or negatively (towards the ''Not expressed'' output) by various features (sorted in descending order of importance) and where each leap represents the SHAP value associated to a given feature and snoRNA. The model output value is in probability for the Logistic Regression (a probability below 0.5 being associated to the ''Not expressed'' label, whereas a probability above 0.5 is associated to the ''Expressed'' label).

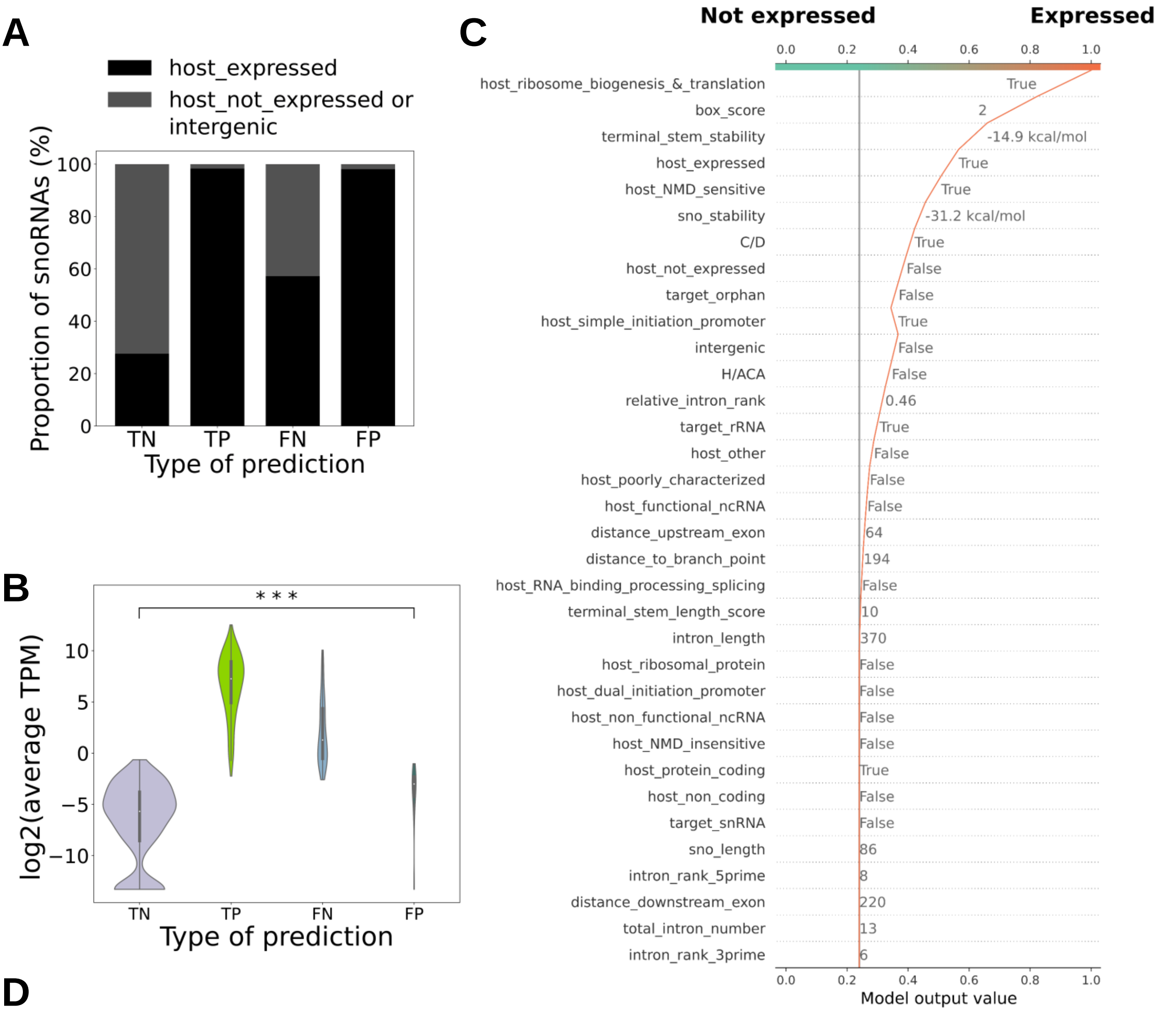

D

**SnoRNA name**	*SNORA22B*	*SNORD86*	*SNORD175*	*Cluster_584*	*SNORD170*	*SNORD132*	*SNORD173*	*Cluster_588*	*AC127002.1*
**SnoRNA type**	H/ACA	C/D	C/D	H/ACA	C/D	C/D	C/D	C/D	H/ACA
**Abundance in Humanref samples (TPM)**	3.69	2.88	2.03	2.29	1.57	1.22	1.36	1.24	1.13

Figure S15 (Supplementary to Figures 3 and 5). Most snoRNAs misclassified as false positives, such as *SNORD86*, are embedded within an expressed host gene and are more expressed than the true negatives snoRNAs. (**A**) Bar chart showing the proportion of snoRNAs embedded in an expressed host gene based on their type of prediction (TN, TP, FN, FP being respectively true negatives, true positives, false negatives and false positives). (**B**) Violin plot showing the average abundance across tissues (log2 of the transcript per million (TPM)) per type of prediction. The TN and FP distributions are significantly different at ***$p < 2 \times 10^{-10}$ (Mann-Whitney *U* test). (**C**) Decision plot showing the ''decision process'' of the Support Vector Machine classifier when predicting *SNORD86* as a FP snoRNA. Each feature value is represented next to the line. (**D**) Potential interesting snoRNAs such as *SNORD86* that were classified as FP in human tissues, but that are expressed (average sample > 1 TPM) HumanRef samples (Nottingham et al., 2016).

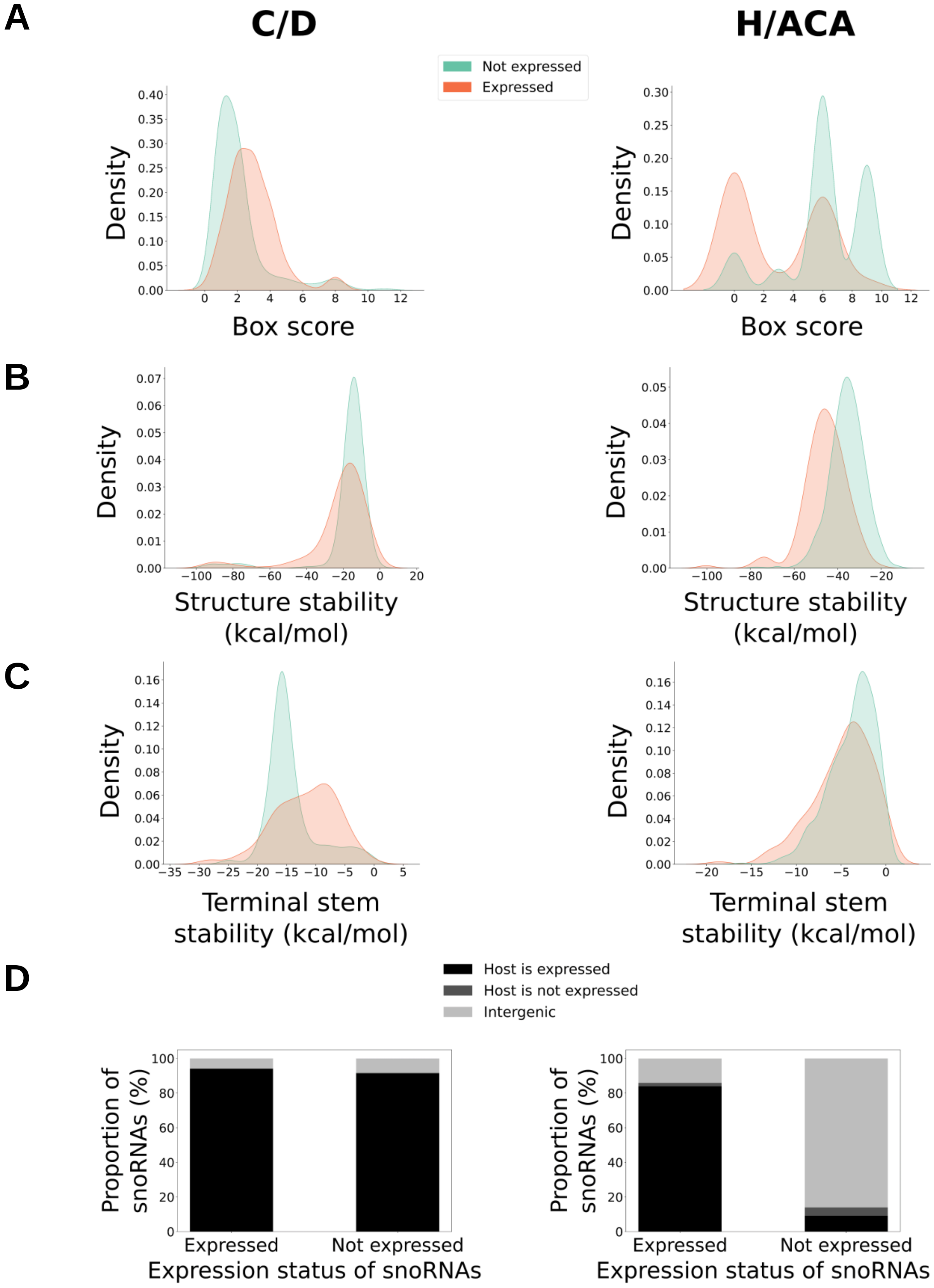

Figure S16 (Supplementary to Figure 6). Distribution of the four features used for snoRNA expression status prediction in mouse for C/D and H/ACA box snoRNAs. (**A-C**) Distribution of C/D (left panel) and H/ACA box (right panel) mouse snoRNAs features such as their box score (**A**), their structure stability (**B**) and their terminal stem stability (**C**), depending on their expression status. (**D**) Bar charts displaying the proportion of snoRNAs per expression status for C/D (left panel) and H/ACA box (right panel) snoRNAs according to their host gene expression level.

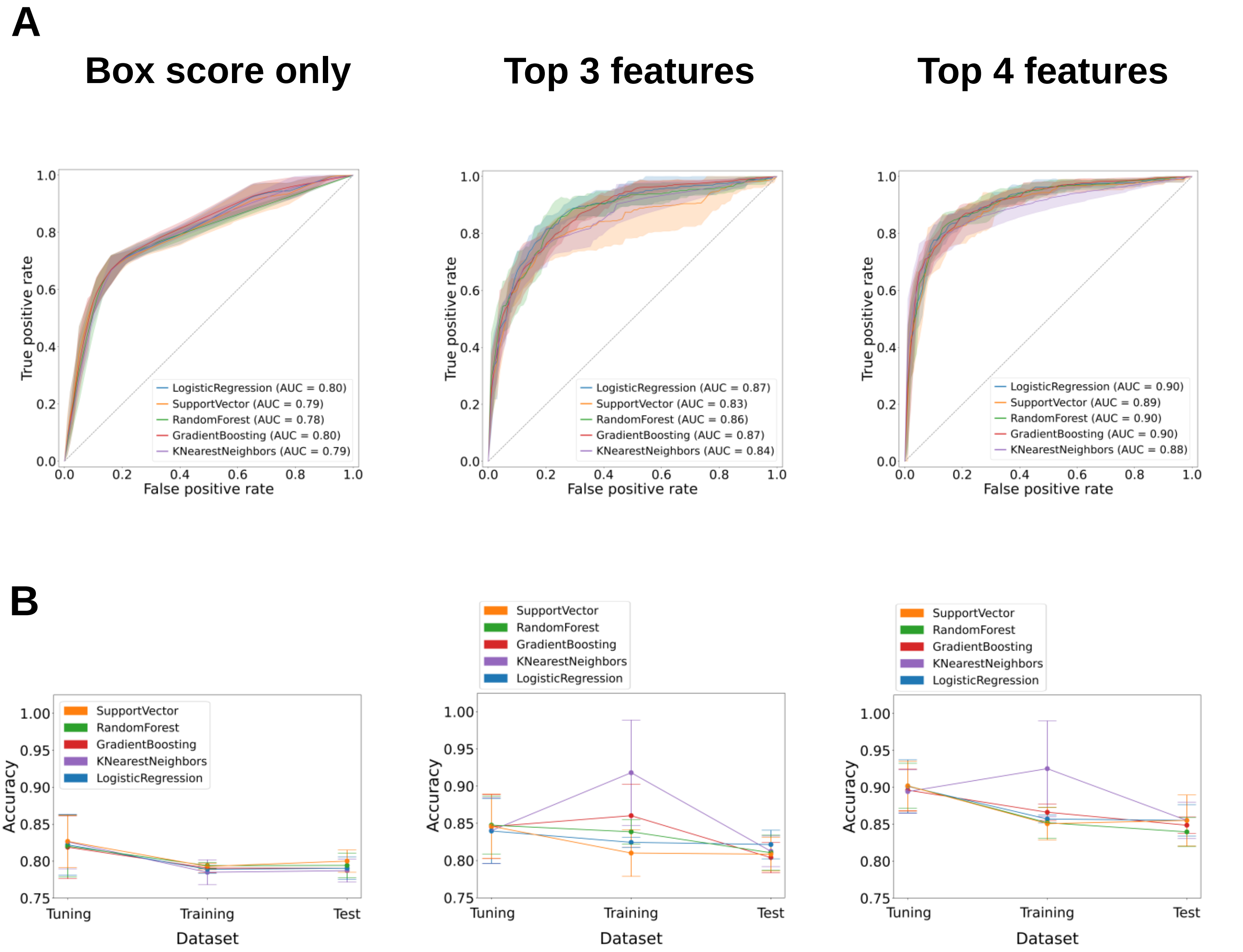

Figure S17 (Supplementary to Figure 3). Models trained only with the most predictive feature (box score) or with the top 3 most predictive feature (box score, sno_stability, terminal_stem_stability) show reasonable performance but that is still lower than models trained with all features or with the top 4 most predictive feature (box score, sno_stability, terminal_stem_stability and host_expressed). (**A**) Receiver operating characteristic (ROC) curves showing the average true and false positive rates of all models on the test dataset across the 10 iterations based on datasets containing only the box consensus score feature (left panel), the top 3 most predictive features (middle panel) or the top 4 most predictive features (right panel). The colored areas above and below each curve represent ± 1 standard deviation for each classifier. The average area under the curve (AUC) is shown for the Logistic Regression, Support Vector Machine, Random Forest, Gradient Boosting and *K*-Nearest Neighbors classifiers. (**B**) Scatter plots showing the average accuracies (± standard deviation) of all models on the tuning, training and test datasets across the 10 iterations based on datasets containing only the box score feature (left panel), the top 3 most predictive features (middle panel) or the top 4 most predictive features (right panel).

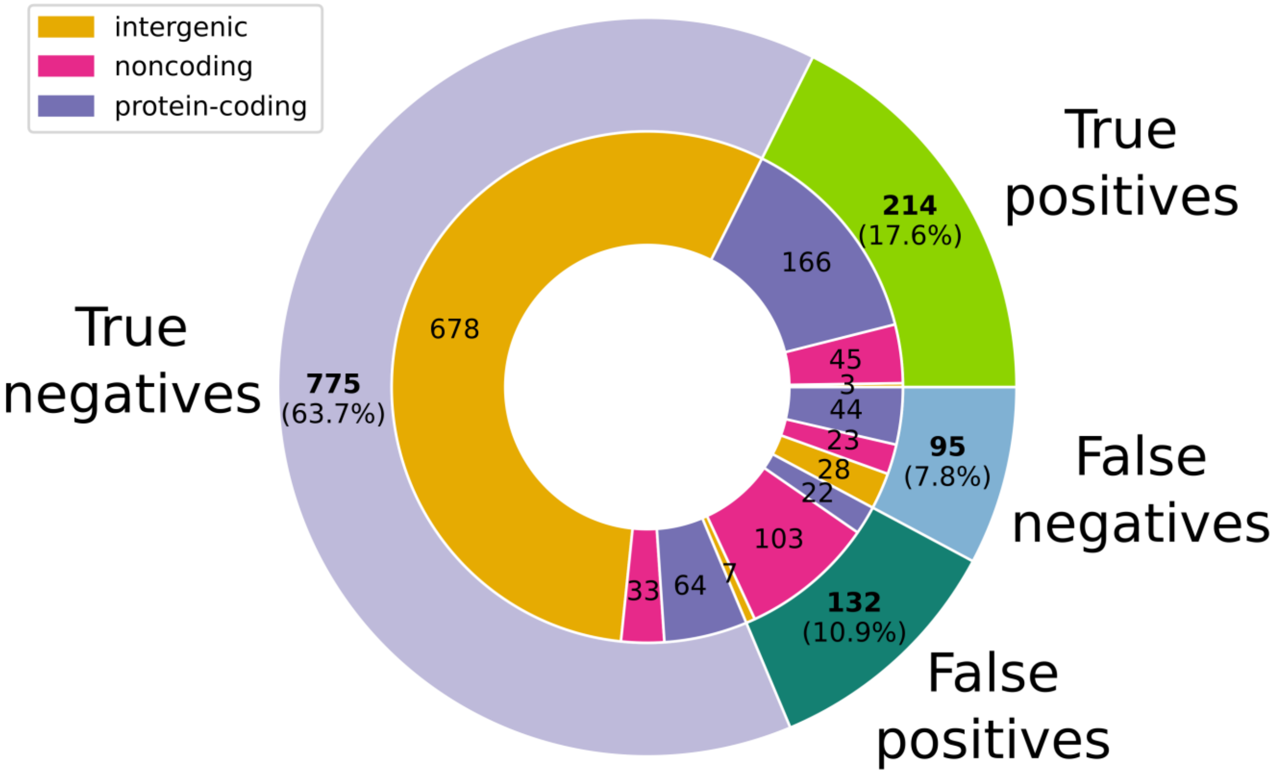

Figure S18 (Supplementary to Figure 6). The Logistic Regression classifier accurately predicts the expression status of mouse snoRNAs. Donut chart showing the number and proportion of true/false positives/negatives (outer donut) and the genomic context of these predicted mouse snoRNAs (inner donut).

Table S2 (Supplementary to Figure 5). Distribution of snoRNA type and host gene biotype for all snoRNAs predicted to be expressed or not expressed across vertebrate species.

Species name	Predicted label	snoRNA type		Host gene biotype			Total number of snoRNAs
		C/D	H/ACA	protein-coding	noncoding	intergenic	
Bos taurus	Expressed	126 (50.4%)	124 (49.6%)	199 (79.6%)	32 (12.8%)	19 (7.6%)	250
	Not expressed	141 (35.2%)	259 (64.8%)	82 (20.5%)	17 (4.2%)	301 (75.2%)	400
Danio rerio	Expressed	45 (45.9%)	53 (54.1%)	83 (84.7%)	12 (12.2%)	3 (3.1%)	98
	Not expressed	99 (78.6%)	27 (21.4%)	46 (36.5%)	17 (13.5%)	63 (50.0%)	126
Gallus gallus	Expressed	73 (56.2%)	57 (43.8%)	116 (89.2%)	7 (5.4%)	7 (5.4%)	130
	Not expressed	44 (71.0%)	18 (29.0%)	30 (48.4%)	1 (1.6%)	31 (50.0%)	62
Gorilla gorilla	Expressed	103 (53.6%)	89 (46.4%)	159 (82.8%)	5 (2.6%)	28 (14.6%)	192
	Not expressed	224 (69.6%)	98 (30.4%)	52 (16.1%)	0 (0.0%)	270 (83.9%)	322
Macaca mulatta	Expressed	211 (66.1%)	108 (33.9%)	206 (64.6%)	46 (14.4%)	67 (21.0%)	319
	Not expressed	299 (58.1%)	216 (41.9%)	107 (20.8%)	33 (6.4%)	375 (72.8%)	515
Ornithorhynchus anatinus	Expressed	89 (14.4%)	530 (85.6%)	570 (92.1%)	15 (2.4%)	34 (5.5%)	619
	Not expressed	84 (1.9%)	4244 (98.1%)	258 (6.0%)	15 (0.3%)	4055 (93.7%)	4328
Oryctolagus cuniculus	Expressed	79 (40.3%)	117 (59.7%)	166 (84.7%)	10 (5.1%)	20 (10.2%)	196
	Not expressed	271 (47.8%)	296 (52.2%)	83 (14.6%)	4 (0.7%)	480 (84.7%)	567
Pan troglodytes	Expressed	101 (49.0%)	105 (51.0%)	177 (85.9%)	1 (0.5%)	28 (13.6%)	206
	Not expressed	284 (49.9%)	285 (50.1%)	89 (15.6%)	0 (0.0%)	480 (84.4%)	569
Rattus norvegicus	Expressed	87 (39.7%)	132 (60.3%)	205 (93.6%)	5 (2.3%)	9 (4.1%)	219
	Not expressed	303 (21.3%)	1119 (78.7%)	151 (10.6%)	31 (2.2%)	1240 (87.2%)	1422
Xenopus tropicalis	Expressed	61 (50.0%)	61 (50.0%)	115 (94.3%)	0 (0.0%)	7 (5.7%)	122
	Not expressed	109 (72.2%)	42 (27.8%)	38 (25.2%)	0 (0.0%)	113 (74.8%)	151
